# The innate immune protein calprotectin promotes *Pseudomonas aeruginosa* and *Staphylococcus aureus* interaction

**DOI:** 10.1038/ncomms11951

**Published:** 2016-06-15

**Authors:** Catherine A. Wakeman, Jessica L. Moore, Michael J. Noto, Yaofang Zhang, Marc D. Singleton, Boone M. Prentice, Benjamin A. Gilston, Ryan S. Doster, Jennifer A. Gaddy, Walter J. Chazin, Richard M. Caprioli, Eric P. Skaar

**Affiliations:** 1 Tennessee Valley Healthcare Systems, US Department of Veterans Affairs, Nashville, Tennessee 37212, USA; 2 Department of Pathology, Microbiology and Immunology, Vanderbilt University School of Medicine, Nashville, Tennessee 37232, USA; 3 Department of Chemistry, Vanderbilt University, Nashville, Tennessee 37232, USA; 4 Department of Medicine, Vanderbilt University School of Medicine, Nashville, Tennessee 37232, USA; 5 Department of Biochemistry, Vanderbilt University School of Medicine, Nashville, Tennessee 37232, USA

## Abstract

Microorganisms form biofilms containing differentiated cell populations. To determine factors driving differentiation, we herein visualize protein and metal distributions within *Pseudomonas aeruginosa* biofilms using imaging mass spectrometry. These *in vitro* experiments reveal correlations between differential protein distribution and metal abundance. Notably, zinc- and manganese-depleted portions of the biofilm repress the production of anti-staphylococcal molecules. Exposure to calprotectin (a host protein known to sequester metal ions at infectious foci) recapitulates responses occurring within metal-deplete portions of the biofilm and promotes interaction between *P. aeruginosa* and *Staphylococcus aureus.* Consistent with these results, the presence of calprotectin promotes co-colonization of the murine lung, and polymicrobial communities are found to co-exist in calprotectin-enriched airspaces of a cystic fibrosis lung explant. These findings, which demonstrate that metal fluctuations are a driving force of microbial community structure, have clinical implications because of the frequent occurrence of *P. aeruginosa* and *S. aureus* co-infections.

## Abstract (web summary)

Co-infections with *Pseudomonas aeruginosa* and *Staphylococcus aureus* are common in cystic fibrosis patients. Here, the authors show that metal depletion induced by a host protein, calprotectin, promotes co-existence of both pathogens by inhibiting production of anti-staphylococcal molecules by *P. aeruginosa*.

## Graphical abstract

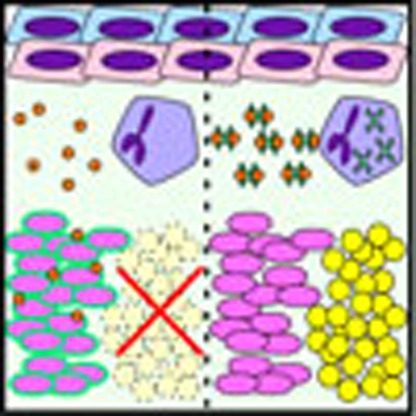

Biofilms are multicellular microbial communities that represent the most common lifestyle of many microorganisms^1^. The unique architecture of biofilms allows these microbial structures to persist in a wide range of niches^2^,^3^. A biofilm can originate from a single microbial species or from numerous and diverse microorganisms, potentially encompassing multiple domains of life^1^,^4^. Importantly, even in biofilms containing a single species, individual cells within different regions of the structure exhibit distinct genetic programmes^5^,^6^. The presence of these spatially-defined regions of physiologically-differentiated cells gives a biofilm tissue-like properties in which different subpopulations of cells serve defined roles in the microbial community to promote the overall health of the biofilm^7^,^8^. It is believed that nutrient gradients that form as the biofilm thickens influence the physiological differentiation of specific loci within the microbial structure^6^,^9^.

Understanding biofilm architecture is important to human health as many infections are caused by biofilms arising from either a single species or a community of pathogens^1^. The role of nutrient metal fluctuations in the formation of these communities is particularly important in the context of infection, owing to the fact that nutritional immunity is one of the major innate defense mechanisms of the host^10^. Nutritional immunity refers to the process by which the host sequesters essential nutrients from invading pathogens. Some of the best studied strategies of nutritional immunity are the chelation of iron (Fe) through the action of host-derived proteins such as transferrin and lactoferrin, as well as chelation of zinc (Zn) and manganese (Mn) by calprotectin^10^,^11^.

Consistent with the theory that host-imposed nutrient starvation can affect microbial community structure, environmental Fe levels impact the gross morphology of various types of single-species biofilms^12^,^13^. Fe levels also influence the composition of pathogenic polymicrobial communities^14^,^15^,^16^. For example, in response to Fe depletion, *Pseudomonas aeruginosa* upregulates production of alkyl hydroxyquinolones (AQs) to lyse staphylococcal cells for use as an Fe source^14^,^17^.

Because of the role nutrient metal levels play in shaping overall microbial community structure, we hypothesized nutrient metal gradients within a developing biomass could elicit dramatic changes in the physiology of bacterial subpopulations. Techniques such as RNA fluorescence *in situ* hybridization (RNA FISH) and reporter gene fusions have been successfully employed in multiple studies to establish the existence of bacterial subpopulations within a biomass^6^,^18^. However, these methods are limited by the number of differentially-expressed targets that can be assessed at one time and require prior knowledge of molecules of interest. Therefore, for the present study we employed a combination of innovative imaging mass spectrometry (IMS) technologies, matrix-assisted laser desorption/ionization (MALDI) IMS^19^ and laser ablation inductively coupled plasma (LA-ICP) IMS, to visualize heterogeneity of proteins, small molecules and metals within a *P. aeruginosa* biofilm in intricate detail.

MALDI IMS does not require prior knowledge of the molecular targets of interest or any molecular tagging mechanisms^19^, making it advantageous over existing technologies employed to study heterogeneous protein expression patterns within a biofilm. This technology employs a matrix homogeneously applied to thinly sectioned samples and a laser to aid in the desorption/ionization of analytes at defined *x*/*y* coordinates. A mass spectrum is collected at each coordinate and the resulting data can be collated to generate heat maps of ion images based on intensity^20^. Ion images reveal the spatial distributions of analytes within the sample^21^,^22^. Using MALDI IMS, hundreds of molecular signals can be detected simultaneously.

LA-ICP IMS is a technology enabling *in situ* analysis of elemental distribution within a two-dimensional sample^23^. Similar to the MALDI IMS workflow, samples are thinly sectioned and mounted onto slides for analysis. A ultraviolet laser is used to systematically ablate material into a sheath gas, which carries the material to the ICP torch for ionization^24^. By visualizing elemental gradients within biofilms, portions of the biomass with differential access to nutrient metals can be identified. The nutrient metal localization can then be compared with the protein patterns assessed by MALDI IMS to identify subpopulations of bacteria that correlate with differential nutrient availability.

Here we use MS to study biofilm architecture and show that Zn and Mn gradients correlate with variations in the levels of many differentially-localized proteins within a *P. aeruginosa* biofilm, including repression of anti-staphylococcal factor biosynthetic machinery in the Zn and Mn-limited portions of the biomass. These results are recapitulated in *P. aeruginosa* cultures exposed to the Zn and Mn-chelating immune protein calprotectin. This repression of anti-staphylococcal factor production in the presence of calprotectin promotes *P. aeruginosa* and *Staphylococcus aureus* co-culture. In addition, in a murine model of pneumonia, the presence of this innate immune protein enables increased levels of *P. aeruginosa* and *S. aureus* co-colonization. Finally, microorganisms consistent with the morphologies of *S. aureus* and *P. aeruginosa* are found to co-exist in calprotectin-enriched regions of a cystic fibrosis lung explant, indicating these findings may have clinical implications regarding our understanding of polymicrobial interactions at the host–pathogen interface.

## Results

### DFR biofilms have distinct structural features

**Figure 1:**
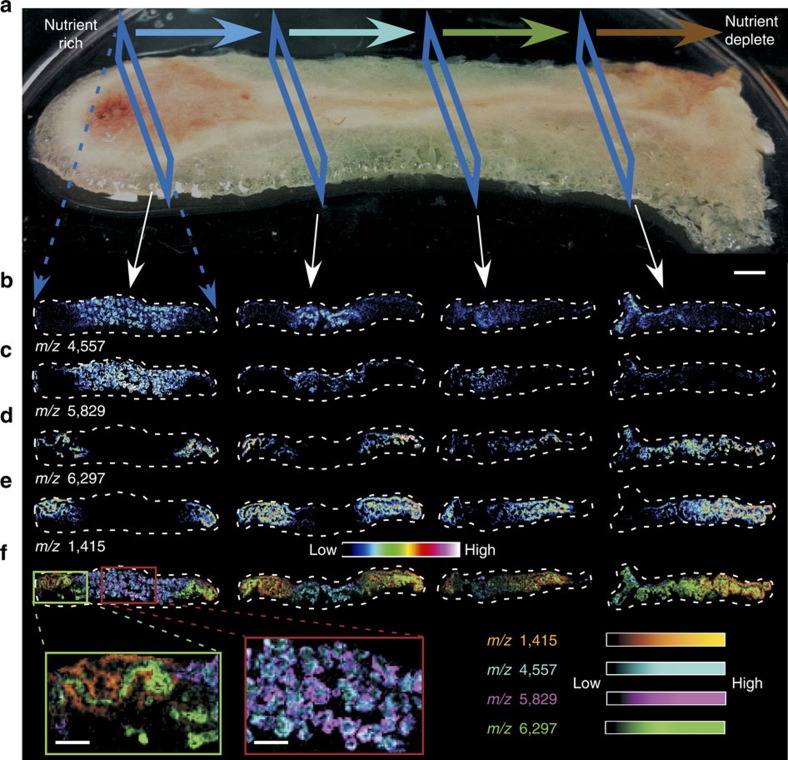
Heterogeneous structure of a *Pseudomonas aeruginosa* biofilm grown in a DFR. (**a**) Representative image of a *P. aeruginosa* biofilm grown in a drip flow reactor (DFR). The flow of nutrients is depicted by the topmost arrows ranging in colour from blue to brown. Blue boxes denote the approximate regions from which 12-μm-thick sections were obtained for MALDI IMS analysis. (**b**,**c**) MALDI IMS signals with differential biofilm localization found primarily in portions of the biomass presumed to be nutrient-replete. (**d**,**e**) MALDI IMS signals primarily localized to portions of the biomass predicted to be nutrient-deplete. (**f**) Overlay of signals shown in **b**–**e** highlighting sublocalization of signals within the predicted nutrient-replete and nutrient-deplete niches with zoomed insets highlighted. Scale bar, 3 mm (inset scale bars, 1 mm).

A drip flow reactor (DFR) generates robust bacterial biofilms^25^. In this system, a continuous influx of nutrients is supplied to a developing biofilm over the course of several days while the waste is removed by gravity flow to minimize shear force (Supplementary Fig. 1a). The biofilms in a DFR form on a provided surface, such as a glass slide. After 6 days of growth in this system, *P. aeruginosa* developed into a robust biofilm that was ∼3-mm thick and capable of withstanding significant physical manipulation. The biofilm structure was visibly influenced by the flow of the nutrient source over the slide surface. The portion of the biomass that developed around the initial drops of medium entering the chamber formed a distinct pore (Supplementary Fig. 1b). As the medium flowed down the length of the slide, a pink-pigmented population of cells formed on this presumably nutrient-rich channel. The biomass forming on the edges of the glass slide with no direct access to the nutrients of the central channel relied on nutrient diffusion and was likely experiencing greater levels of starvation. This presumably nutrient-depleted population was visibly distinct from the nutrient-rich central channel (Fig. 1a and Supplementary Fig. 1b). Despite the visible differences in these distinct portions of the *P. aeruginosa* DFR biofilm, all regions of the biomass were composed of approximately equivalent ratios of live to dead cells as determined by live/dead staining confocal microscopy (Supplementary Figs 2 and 3). Therefore, the structural differences apparent throughout the biomass were not associated with alterations in cell viability. In addition, electron microscopy of the biomass did not identify the presence of any contaminating microorganisms contributing to the heterogeneous nature of the DFR biofilm (Supplementary Fig. 2).

### Biofilm heterogeneity can be visualized using MALDI IMS

To identify differential protein expression within the *P. aeruginosa* biofilm, various portions of the biomass both proximal and distal to nutrient influx were analysed using MALDI IMS. This analysis revealed the presence of distinct subpopulations throughout the biomass by tracking unique mass to charge (*m/z*) ratios. Much of this differential protein expression correlated with predicted nutrient gradients (Fig. 1b–e). Many molecules that were abundant in biofilm sections proximal to the influx of nutrients were virtually absent in distal sections (Fig. 1b,c), whereas molecules relegated to the edges of the presumably nutrient-rich sections were enriched in the distal portions of the biomass (Fig. 1d,e). When differentially-expressed *m/z* signals were overlaid, there were distinct subpopulations within the nutrient-rich and nutrient-deplete portions of the biofilm, indicating MALDI IMS is capable of uncovering biofilm heterogeneity extending beyond that intuitively dictated by nutrient gradients (Fig. 1f). Intricate details in biofilm architecture revealed by MALDI IMS include a heterogeneous population of cells within the central channel (*m/z* 4,557 and 5,829, Fig. 1f) and the nutrient-deplete edges (*m/z* 1,415 and 6,297, Fig. 1f). A small population of cells residing at the interface of the central channel and nutrient-deplete edges was also isolated (*m/*z 9,731, Supplementary Fig. 4a). Additional signals were found primarily at the air-exposed surface of the biofilm while others were only present deep within the portions of the biomass adhered to the glass slide. These trends in biofilm heterogeneity may be influenced by oxygen availability as oxygen might not efficiently diffuse throughout the ∼3-mm thick biomass (*m/z* 5,867 and 11,701, Supplementary Fig. 4b). This finding is consistent with similar results identifying differential gene expression within anoxic regions of biofilms^5^,^18^,^26^. In total, these results demonstrate that MALDI IMS is a useful tool to study biofilm heterogeneity, capable of determining the spatial localization of hundreds of proteins in a single experiment.

### Identification of differentially-expressed biofilm protein*s*

**Figure 2:**
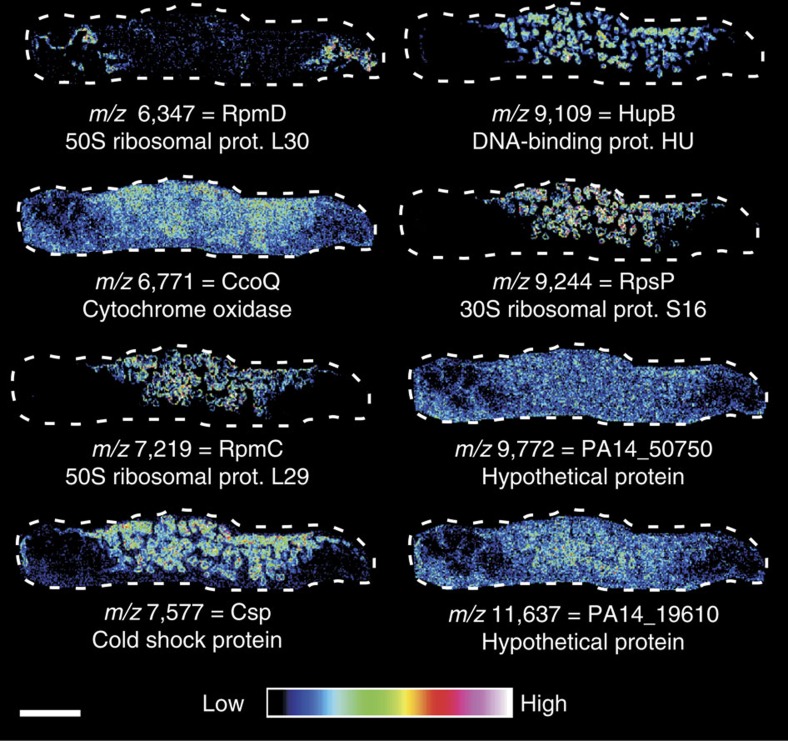
MALDI IMS signals identified using bottom-up proteomics. *m/z* values and associated protein designations are included adjacent to MALDI IMS images of the biofilm section proximal to the nutrient pore highlighted in Fig. 1. These identifications derive from signals that were reproducibly detected during the analysis of 15 replicate DFR biofilms. Scale bar, 3 mm.

MALDI IMS analysis of replicate biofilms revealed numerous ions with consistent and reproducible patterns within the biomass. These *m/z* species were targeted for identification using bottom-up proteomics approaches. Initial identification strategies revealed a number of small ribosomal proteins, RpmC, RpmD and RpsP, are either enriched in the central channel or the putatively nutrient-deplete edge of the biofilm (Fig. 2). Ribosomal proteins can be regulated in response to Zn levels^27^; therefore, it is possible these ribosomal proteins are differentially-regulated in response to nutrient gradients occurring within the biomass. Proteins of unknown function, including the proteins PA14_19610 and PA14_50750, as well as proteins related to general stress, such as cold shock protein Csp were also identified. In addition, general metabolic proteins were detected, including cytochrome cbb oxidase CcoQ and DNA-binding protein HupB. Many of these signals appeared to be distributed relatively evenly throughout the biomass with subtle decreases in signal intensity in the biofilm edge likely attributed to decreased cell density in regions with limited nutrient access. These results demonstrate MALDI IMS signals can be identified using bottom-up proteomic approaches.

### Identification of metal gradients in biofilms

**Figure 3:**
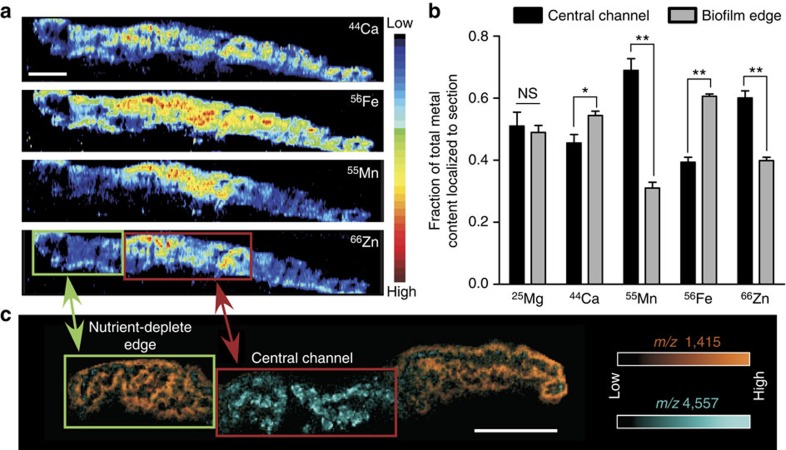
Differences in metal distribution patterns revealed by LA-ICP IMS correlate with differential protein localization within the biofilm. (**a**) LA-ICP IMS analysis of a biofilm section containing a defined ‘central channel' and ‘nutrient-deplete edge'. Scale bar, 2 mm. (**b**) ICP-MS quantification of metal levels within distinct portions of the biofilm. Error bars represent s.d. of data derived from triplicate biofilms. ‘*' designates *P*<0.05, ‘**' designates *P*<0.002 as determined by a Student's *t*-test. (**c**) MALDI IMS analysis highlights the presence of differential protein distribution patterns that follow similar trends to the metal localization patterns. Scale bar, 3 mm. NS, not significant.

Alterations in metal availability have been shown to influence overall biofilm architecture^12^,^13^. In an effort to determine which metals are restricted in the nutrient-deplete edges of the *P. aeruginosa* DFR biofilm, a section of biomass was analysed using LA-ICP IMS. This analysis revealed that not all metals were equally depleted within the biofilm edges. Zn and Mn were found in low levels in the biofilm edge; however, Fe and Ca were distributed throughout the biomass (Fig. 3a). To verify that the visibly distinct edges of the *P. aeruginosa* biofilm corresponded to regions experiencing Zn and Mn deprivation, the nutrient-deplete edges of the DFR biofilm were dissected away from the central channel (Supplementary Fig. 5a). These samples were homogenized, digested and subjected to ICP-MS analysis to determine total metal concentration, revealing that the nutrient-deplete edges of the DFR biofilm indeed correspond to areas of low Zn and Mn levels (Fig. 3b; Supplementary Fig. 5b). Interestingly, when the measured metal concentrations were adjusted to total protein content of the sample, an apparent increase in Fe and Ca was observed in the biofilm edge. These metal localization patterns correspond well with regions of differential protein production (Fig. 3c). These results indicate nutrient metals differentially diffuse throughout the bacterial biomass.

### Proteomic analysis of Zn- and Mn-deplete portions of biofilms

**Figure 4:**
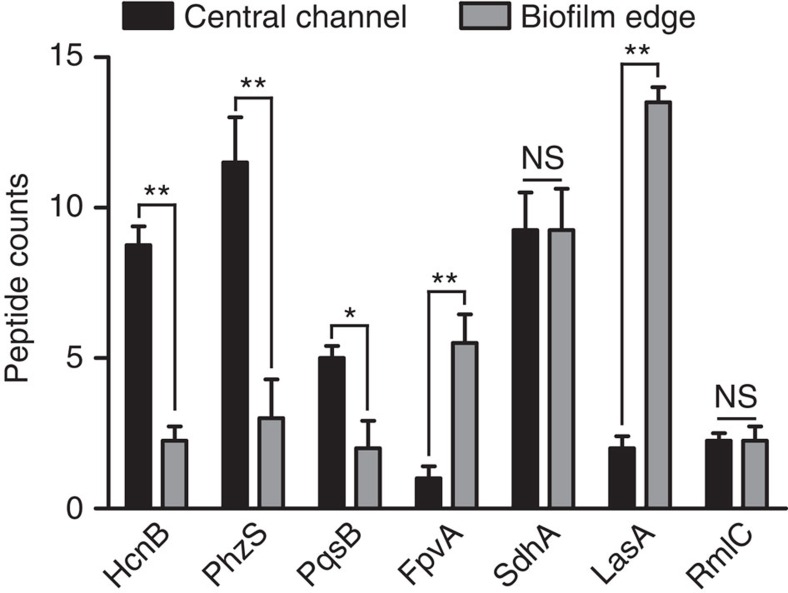
Bottom-up proteomics reveals repression of anti-staphylococcal biosynthetic proteins in the edge of the *P. aeruginosa* biofilm. HcnB, PhzS and PqsB are components of various anti-staphylococcal biosynthetic pathways that were found in lower abundance in the biofilm edge. While expression of the corresponding genes has been shown to be both Fe and quorum sensing (QS)-regulated, the proteins' distribution profiles did not correlate with those of known Fe-responsive proteins such as FpvA and SdhA or proteins encoded by known QS-responsive genes such as LasA and RmlC. Error bars are s.e.m. derived from biological triplicate samples processed in four technical replicates per biofilm. ‘*' denotes *P*<0.05, ‘**' denotes *P*<0.02 as determined by a Student's *t*-test. NS, not significant.

The dissected biofilm samples were submitted for bottom-up proteomic analysis to augment the MALDI IMS studies, and to identify overall protein changes that occur in response to the nutrient metal-depletion found in the biofilm edge. Overall, the detected proteins in the biofilm edge and central channel were largely similar with several distinct changes in abundance (Supplementary Data 1–3). One trend of note was the suppression of several well-studied biosynthetic enzymes of *P. aeruginosa* in the nutrient-deplete biofilm edge (Fig. 4; Supplementary Data 2). The *hcn*, *phz* and *pqs* genes control production of hydrogen cyanide, pyocyanin and AQs, respectively. Each of these molecules has antimicrobial capabilities that enable *P. aeruginosa* to outcompete surrounding bacterial species^28^. *S. aureus*, an organism commonly found in polymicrobial infections involving *P. aeruginosa,* is particularly susceptible to these products. These biosynthetic operons are regulated by quorum sensing (QS)^29^. However, overall QS capabilities were not inhibited in the biofilm edge as the QS-activated enzyme LasA was dramatically upregulated in this niche, while other QS-regulated proteins such as RmlC were unchanged throughout the biomass (Fig. 4). In addition to QS-based regulation, the anti-staphylococcal activity of *P. aeruginosa* is increased under Fe-limiting conditions^14^. However, in the Zn and Mn-limited portion of the biofilm, *P. aeruginosa* appears to repress the production of these factors. The repression of anti-staphylococcal factor production in the biofilm edge contrasts with other proteins known to be activated under Fe-limitation such as the pyoverdine receptor FpvA^30^, which was enriched in the biofilm edges, or the Fe-regulated succinate dehydrogenase SdhA^31^, which was constitutively detected throughout the biomass (Fig. 4; Supplementary Data 3). Because FpvA was enriched and HcnB, PhzS and PqsB were repressed in a Zn- and Mn-deplete yet Fe-replete portion of the biofilm, it is possible that a subset of characterized Fe-regulated genes is responsive to multiple metal ions.

### Calprotectin represses production of antimicrobial compounds

To determine which of the various genetic changes observed in the nutrient-deplete biofilm edge can be specifically attributed to Zn and/or Mn deprivation, *P. aeruginosa* biofilms were treated with calprotectin to induce Zn and Mn starvation throughout the biomass. Calprotectin is an abundant innate immune protein with antimicrobial activity deriving from its ability to sequester transition metals away from invading pathogens^32^,^33^. Proteomic analyses indicated that calprotectin diffusion throughout the biomass was incomplete, likely owing to the protective features of the biofilm (Supplementary Fig. 6). However, these studies identified a subset of differentially-localized proteins that were constitutively expressed or repressed throughout the biofilm on calprotectin treatment (Supplementary Figs 7 and 8; Supplementary Data 2 and 3). This subset of proteins, which included many of the anti-staphylococcal biosynthetic enzymes (Supplementary Data 2), may represent the targets most responsive to calprotectin-induced metal starvation. However, the incomplete diffusion of calprotectin throughout the biomass indicated there may be additional calprotectin-dependent responses undetected in this experiment. Therefore, we sought to determine if these metal-dependent responses could be recapitulated in planktonic culture.

**Figure 5:**
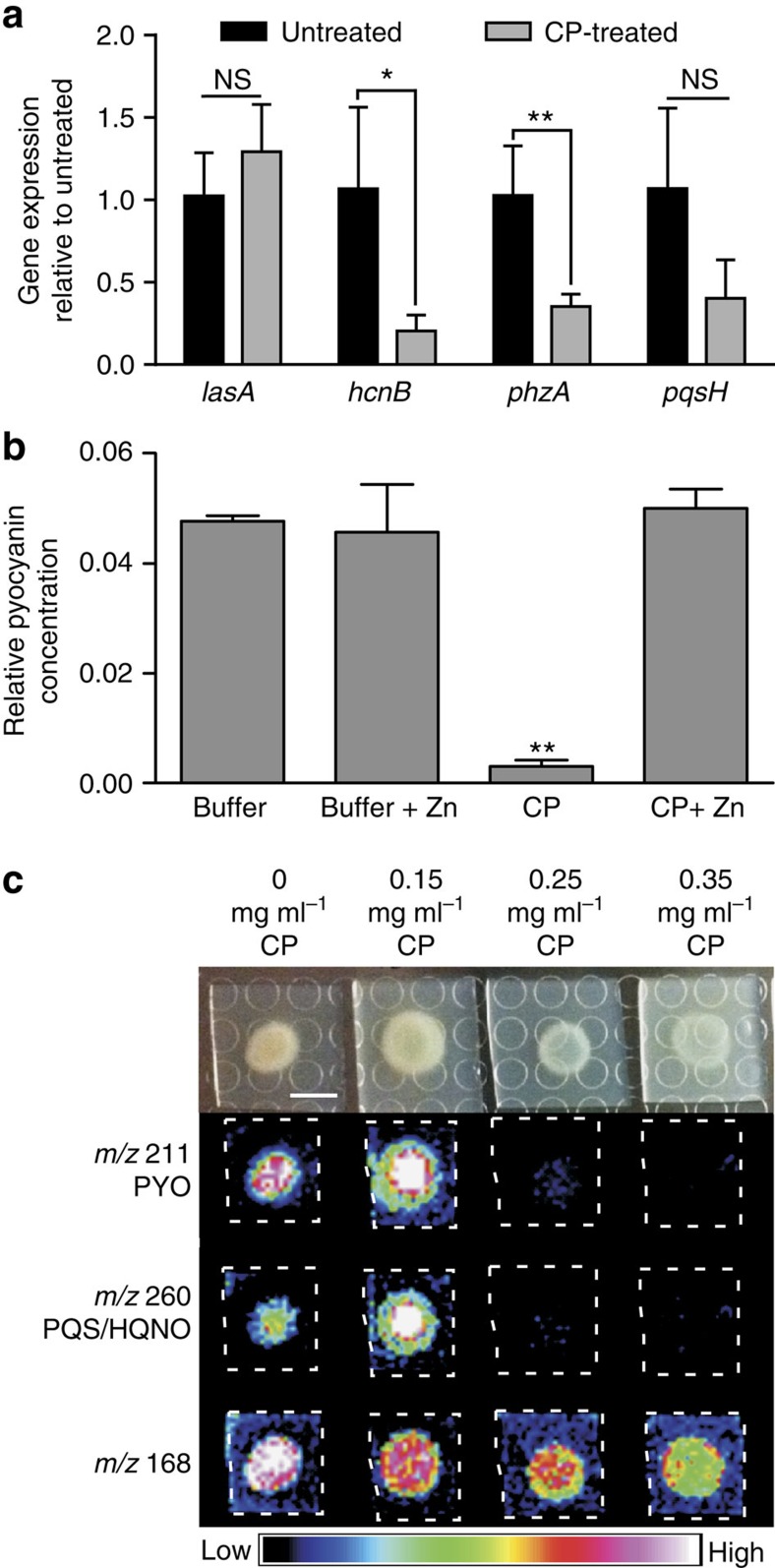
CP-treatment represses *P. aeruginosa* biosynthetic genes responsible for production of numerous anti-staphylococcal factors. (**a**) qRT–PCR quantification of anti-staphylococcal biosynthetic gene transcripts in the presence or absence of 0.25 mg ml^−1^ calprotectin (CP). (**b**) Quantification of pyocyanin in cultures grown in the presence or absence of 0.25 mg ml^−1^ CP and/or 10 μM Zn. ‘*' designates *P*<0.05, ‘**' designates *P*<0.002 as determined by a Student's *t*-test. Error bars represent s.d. of triplicate samples. (**c**) MALDI IMS detection of secondary metabolites, pyocyanin (PYO) and the alkyl hydroxyquinolones PQS and HQNO, as well as a control ion at *m/z* 168 on media embedded with increasing CP concentrations. Scale bar, 5 mm. NS, not significant.

RNA-seq analysis of planktonic cultures grown in the presence or absence of calprotectin revealed additional metal-dependent genetic responses (Supplementary Data 4 and 5). Quantitative reverse transcription–PCR (qRT–PCR) quantification of anti-staphylococcal biosynthetic gene expression revealed significant repression of pyocyanin and hydrogen cyanide biosynthetic genes on calprotectin exposure in planktonic culture with trends towards decreasing levels of 2-heptyl-3-hydroxy-4-quinolone (PQS) biosynthetic gene expression (Fig. 5a). The unchanging levels of *lasA* transcription provided evidence that these responses did not result from decreased QS capacity of the culture. These transcriptional responses corresponded with a dramatic reduction in pyocyanin production that was reversed by the addition of exogenous Zn to the cultures (Fig. 5b). The metal-dependent regulation of anti-staphylococcal secondary metabolites was also visualized using MALDI IMS of bacteria grown on agar plates infused with increasing levels of calprotectin. MALDI IMS identified signals with *m/z* ratios corresponding to pyocyanin and the AQs, PQS and 4-hydroxy-2-heptylquinoline-*N*-oxide (HQNO), which decreased with increasing calprotectin concentrations (Fig. 5c). These signals were confirmed to be pyocyanin and HQNO/PQS as they were absent in strains with mutations in these biosynthetic operons (Supplementary Fig. 9). In total, these data demonstrate that calprotectin-induced metal deprivation significantly reduces anti-staphylococcal factor production under multiple growth conditions in both biofilm and planktonic states.

### Calprotectin promotes *P. aeruginosa* and *S. aureus* co-culture

**Figure 6:**
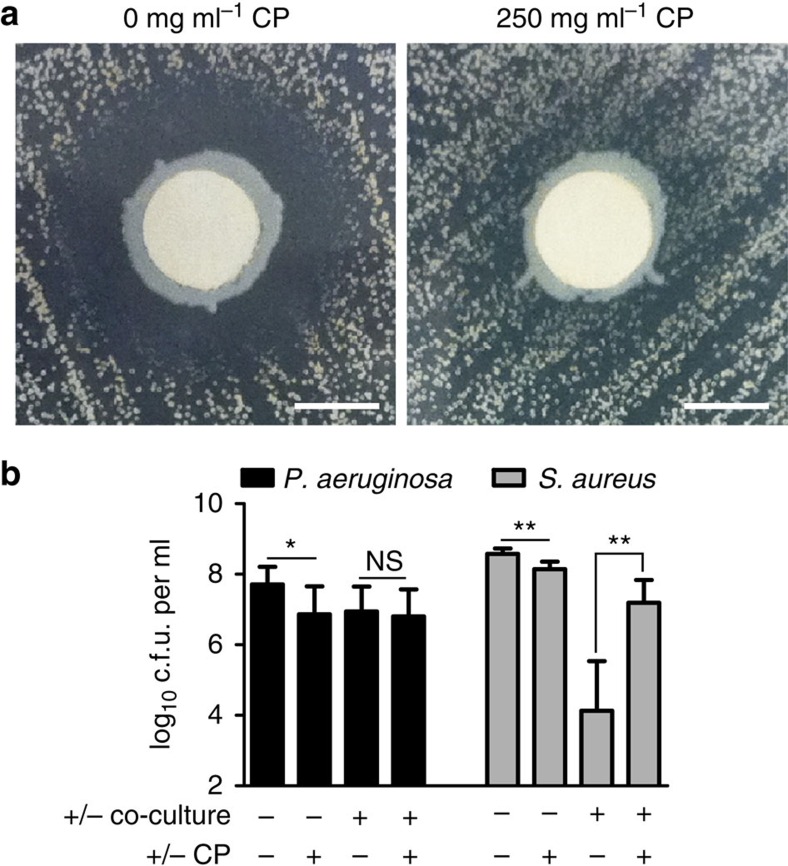
CP exposure promotes microbial interaction between *P. aeruginosa* and *S. aureus*. (**a**) Staphylococcal zones of inhibition on LB agar plates with or without the addition of calprotectin (CP). Lawns of *S. aureus* were spread onto plates and *P. aeruginosa* cultures were spotted onto paper disks. Zones of inhibition were visualized after 24 h of incubation. Scale bars, 5 mm. (**b**) Colony forming unit (c.f.u.) obtained after liquid growth with or without 0.25 mg ml^−1^ CP. Error bars represent s.d. of replicate experiments from nine separate days. Experiments were performed in biological triplicate on each day. ‘*' designates *P*<0.02, ‘**' designates *P*<0.0002 as determined by a Student's *t*-test. NS, not significant.

The biological impact of the metal-dependent regulation of *P. aeruginosa* anti-staphylococcal factors was tested in co-culture experiments with *S. aureus* under multiple types of growth conditions. In agar-based assays, a lawn of *S. aureus* cells was plated onto the surface of LB agar infused with either calprotectin or calprotectin-free buffer, and *P. aeruginosa* culture was spotted onto discs positioned on the agar surface (Fig. 6a). On calprotectin-free medium, a characteristic zone of clearance in the staphylococcal lawn formed around *P. aeruginosa*. However, in the presence of calprotectin, this zone of clearance was dramatically diminished. In a titration of calprotectin concentrations, maximal inhibition of *P. aeruginosa* anti-staphylococcal activity was shown to occur in the presence of 0.25 mg ml^−1^ calprotectin on both agar plates and in liquid culture (Supplementary Fig. 10). These results were recapitulated in biofilm-based co-culture experiments (Supplementary Fig. 11) and planktonic-based co-culture experiments (Fig. 6b; Supplementary Figs 10b and 12). *P. aeruginosa* viability was minimally impacted by either calprotectin-treatment or co-culture conditions (Supplementary Fig. 12a). However, *S. aureus* survival was significantly impaired by the presence of *P. aeruginosa*. This impaired survival in the presence of *P. aeruginosa* was largely reversed by exposure to calprotectin in the growth medium (Fig. 6). The effect of calprotectin exposure on *S. aureus* and *P. aeruginosa* co-culture was dependent on metal-binding activity of calprotectin as a mutant protein incapable of chelating transition metals^34^ behaved equivalently to calprotectin-free media conditions (Supplementary Fig. 12a). Calprotectin can bind multiple transition metals^32^,^35^; therefore, Fe, Mn and Zn were individually added to co-culture conditions to determine the specific metal starvation signal resulting in repression of anti-staphylococcal metabolite production (Supplementary Fig. 12b). This experiment demonstrated that only the addition of Zn was capable of fully reversing the effects of calprotectin treatment. Zn addition significantly restored anti-staphylococcal activity of *P. aeruginosa* when added at a 1:1 molar ratio with calprotectin and fully restored anti-staphylococcal function when present at a 2:1 molar ratio, saturating the two transition metal-binding sites of calprotectin (Supplementary Fig. 12c). These findings reveal that Zn starvation promotes *P. aeruginosa* and *S. aureus* interaction under a number of different culture conditions.

### Calprotectin promotes co-infection of the murine lung

**Figure 7:**
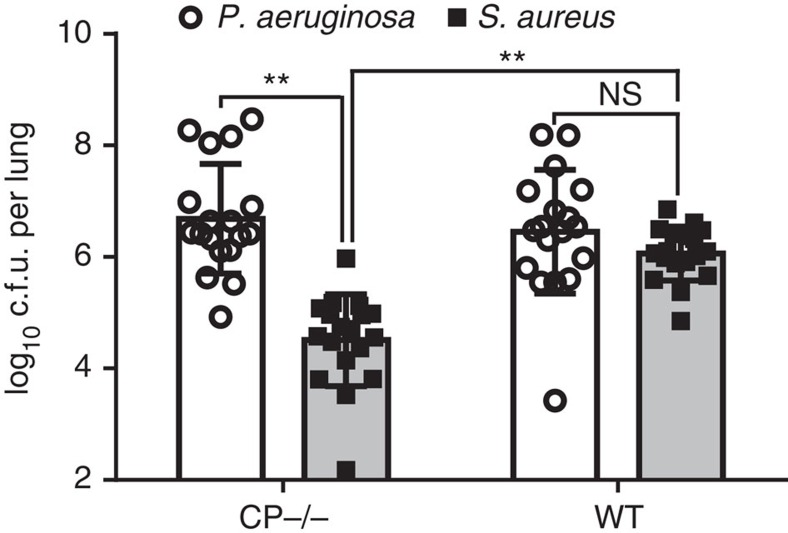
Calprotectin production during infection of the murine lung promotes *S. aureus* and *P. aeruginosa* co-colonization. Wild-type (WT) or calprotectin-deficient (CP^−/−^) C57BL/6 mice were intranasally infected with equivalent amounts of *P. aeruginosa* and *S. aureus*. Bacterial burdens were enumerated 38 h post infection. Equivalent burdens of *P. aeruginosa* were obtained from both mouse strains. In WT mice, *S. aureus* levels were equivalent to the *P. aeruginosa* burden. However, in mice lacking calprotectin production, *P. aeruginosa* significantly outcompeted *S. aureus* during the course of infection. ‘**' designates *P*<0.0000001 as determined by a Student's *t*-test. Error bars represent s.d. of data that was replicated in two independent experiments performed on separate days. *n*=18 mice (shown as individual dots or squares) for both WT and CP^−/−^ groups. NS, not significant.

Because calprotectin-treatment promoted microbial co-culture between *P. aeruginosa* and *S. aureus* under a variety of *in vitro* growth conditions, it seemed possible that this phenomenon might also occur from calprotectin exposure experienced during infection. To test whether or not calprotectin production can promote the establishment of polymicrobial infections, calprotectin-deficient mice were obtained. Co-infections in these mice were compared with co-infections occurring in wild-type C57BL/6 mice, the parental background from which the calprotectin-deficient mouse strain was derived^36^. When calprotectin-deficient mice were intranasally inoculated with equal numbers of *P. aeruginosa* and *S. aureus*, *P. aeruginosa* outcompeted *S. aureus* by 38 h post infection. However, when wild-type C57BL/6 mice were infected with the same inoculum, equivalent numbers of *P. aeruginosa* and *S. aureus* were present at 38 h post infection (Fig. 7). These data demonstrate that the presence of calprotectin can promote co-colonization of the murine lung.

### Polymicrobial lung infections co-localize with calprotectin

**Figure 8:**
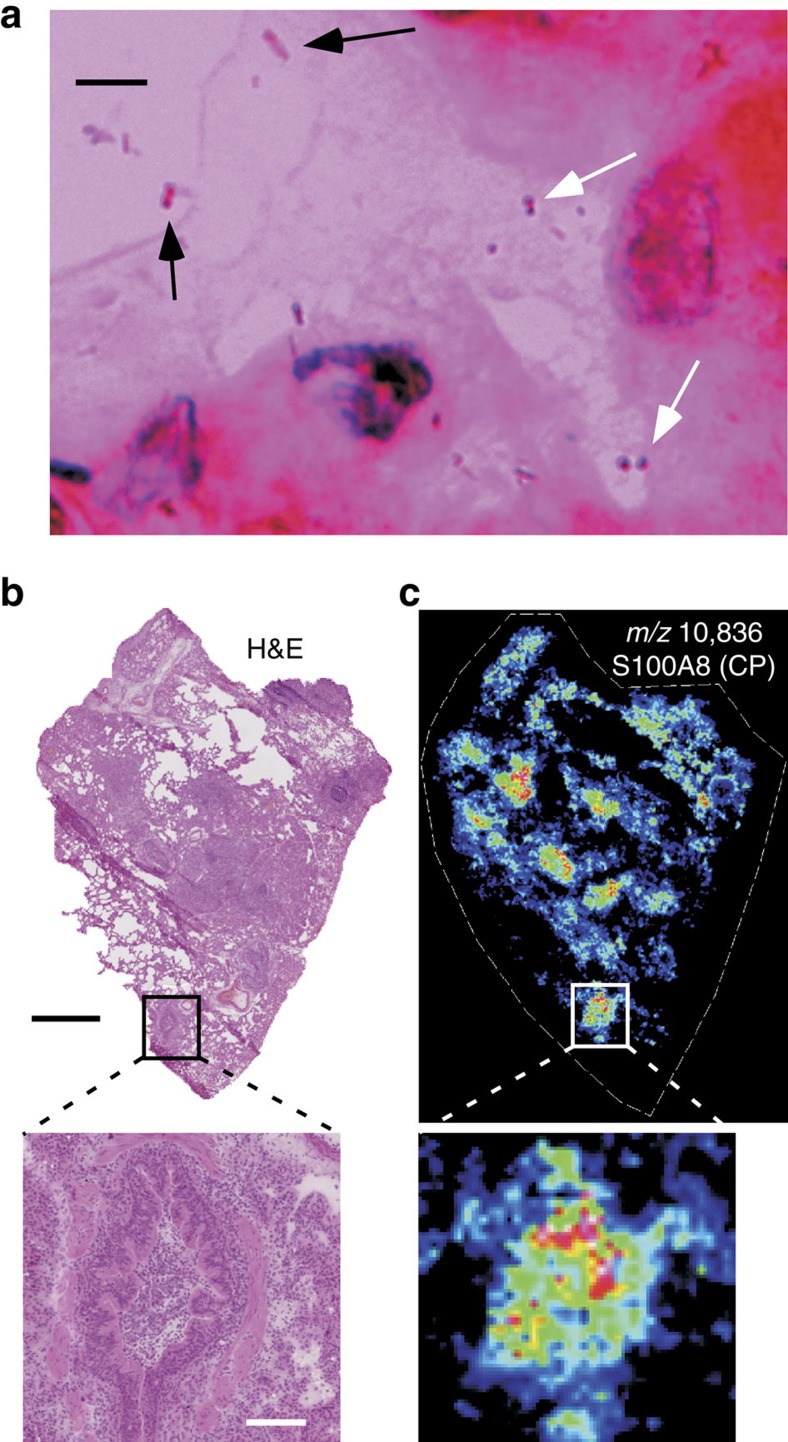
Polymicrobial communities exist within calprotectin-replete airways of a cystic fibrosis lung explant. (**a**) Gram-stain of an inflamed airspace of a cystic fibrosis lung explant containing bacterial morphologies consistent with both *S. aureus* (white arrows) and *P. aeruginosa* (black arrows). Scale bar, 5 μm. (**b**) H&E histology of the cystic fibrosis lung explant with the inflamed airspace that was visualized by Gram-stain enlarged (inset). Black scale bar, 2 mm; inset white scale bar, 200 μm. (**c**) MALDI IMS analysis of the lung explant reveals calprotectin-enrichment at inflammatory foci (inset).

While cystic fibrosis patients commonly become infected with both *P. aeruginosa* and *S. aureus*, it is unknown whether these organisms share common niches within the human lung or remain segregated from each other within specific pulmonary compartments. Optical images were obtained from a human cystic fibrosis lung explant known to be chronically co-infected with *P. aeruginosa* and *S. aureus*. The images revealed that bacteria with morphological and Gram-staining features consistent with *P. aeruginosa* and *S. aureus* occupied the same airspace within the diseased lung (Fig. 8a). The portion of the lung that housed the polymicrobial infection exhibited a high degree of inflammation as determined by haematoxylin and eosin (H&E) staining, as well as an associated accumulation of calprotectin as visualized by MALDI IMS (Fig. 8b,c). Calprotectin accumulation was monitored by following the signal at *m/z* 10,836 indicative of the S100A8 subunit of calprotectin^37^. These results strongly indicate that *P. aeruginosa* and *S. aureus* interactions can occur within a diseased lung and confirm that calprotectin is abundantly present at sites of inflammation and polymicrobial infection within the lungs of patients with cystic fibrosis.

## Discussion

The finding that *P. aeruginosa* can repress its anti-staphylococcal capacity in the presence of an abundant innate immune protein adds to the growing body of literature describing the role of the host environment in shaping the physiology of polymicrobial communities. Previous studies have described host and microbial factors that contribute to the switch from *S. aureus-* to *P. aeruginosa*-dominated infections that occur during the lifetime of many cystic fibrosis patients^16^,^38^. The current manuscript provides insight into the impact of the host environment on the physiology of the stable *P. aeruginosa* and *S. aureus* co-infections that develop in a large population of cystic fibrosis patients. In total, these data provide an additional facet to the complex interactions occurring at the host–pathogen interface during chronic infection.

*P. aeruginosa* and *S. aureus* co-infections are associated with diseases characterized by high levels of inflammation and subsequent calprotectin accumulation such as chronic wound infection and pulmonary infection in cystic fibrosis patients^39^,^40^,^41^. Therefore, the finding that *P. aeruginosa* can dampen its anti-staphylococcal activity in response to calprotectin-induced Zn starvation may have clinical implications regarding the common occurrence of *P. aeruginosa* and *S. aureus* co-infections. This response is surprising given that, under conditions of starvation, it would be advantageous to inhibit the growth of competing organisms. Therefore, an alternative evolutionary explanation for the development of this behaviour must exist. One theory originates from the fact that *S. aureus* has acquired numerous mechanisms to counteract endogenous host defenses^42^. The infection course of cystic fibrosis patients typically begins with *S. aureus* colonization of the lung prior to infection with *P. aeruginosa*, hinting that *P. aeruginosa* might benefit from initial seeding of the lung environment with *S. aureus*^43^. Therefore, it is possible that over the course of chronic infection, *P. aeruginosa* might find it evolutionarily advantageous to maintain a population of *S. aureus* to combat the host immune response. This possibility is supported by recent evidence that *P. aeruginosa* populations benefit from the presence of toxin-producing *S. aureus* strains in both the context of wound and lung infection^40^,^44^. While no benefit for *P. aeruginosa* was apparent in the acute co-infection data presented in the current manuscript, it is possible that the advantages of co-infection might become more evident during chronic infection. The theory that *P. aeruginosa* benefits from polymicrobial infection provides an additional explanation for the occurrence of numerous *P. aeruginosa* mutants with decreased anti-staphylococcal capacity commonly isolated from chronically-infected cystic fibrosis patients^28^,^45^,^46^,^47^. These findings could have clinical ramifications owing to the unique physiology, antibiotic resistance and disease severity associated with polymicrobial communities^48^,^49^.

Through the application of MALDI IMS, we were able to identify many different subpopulations within the microbial structure. Because the host environment at infectious foci is known to contain low levels of bioavailable Zn and Mn, the study of the populations arising within the Zn and Mn-deplete regions of the biomass may provide insight into the physiology of *P. aeruginosa* at the host–pathogen interface^50^,^51^. In addition, MALDI IMS enabled the identification of subpopulations within the nutrient-replete portions of the biomass, as well as populations with no apparent correlation with nutrient gradients. Further study of each of these populations will expand our knowledge of biofilm architecture and provide insight into the functionality of subpopulations in response to various environmental pressures. These studies complement previous research that has utilized MALDI IMS to explore molecular interactions occurring at the interface of competing microbial communities^52^,^53^.

The combinatorial application of MALDI IMS, LA-ICP IMS and proteomics towards the study of biofilm architecture provided insights into metal homeostasis within these microbial communities. For example, Fe siderophore biosynthetic enzymes were dramatically upregulated in the biofilm edge even though Fe was found to be replete in this portion of the biomass (Supplementary Data 3). These results suggest that Zn starvation or a combination of Zn and Mn limitation is capable of increasing siderophore production. In support of this hypothesis, induction of pyoverdine biosynthesis by calprotectin-treatment is completely reversed by the addition of Zn (Supplementary Fig. 13). It is perhaps the increase in siderophore production that is responsible for the enrichment of Fe in the biofilm edge. Interestingly, ICP-MS analysis of the biofilm also revealed that the edges were enriched in calcium, a metal known to provide structural integrity to *P. aeruginosa* biofilms^54^. Therefore, it is possible the subpopulation found in the biofilm edge has an increased requirement for this element. Future studies delving into the Zn- and/or Mn-responsive control of siderophore and antimicrobial compound production in *P. aeruginosa* may provide insights into as-yet-unstudied metalloregulatory mechanisms in numerous other bacterial species.

In total, these data demonstrate that the application of MALDI IMS and LA-ICP IMS to the study of microbial community structure will further our understanding of the process of differentiation within a clonal population, as well as enable studies into the role of nutritional gradients in the development of polymicrobial communities. The identification of multiple subpopulations and numerous differentially-expressed proteins and small molecules in bacterial biofilms using MALDI IMS is a testament to the power of this technology for the study of microbial community structure, as well as the analysis of sample heterogeneity in general. Future applications of MALDI IMS towards the study of biofilm architecture may uncover additional bacterial subpopulations, provide insight into the functionality of these populations, and reveal novel factors driving biofilm differentiation.

## Methods

### Bacterial strains

The *P. aeruginosa* strain used in this study was the highly virulent human wound isolate PA14 (ref. 55). The transposon insertion mutants targeting *phzM* and *pqsC* were part of a transposon mutant library derived from this parental background^56^. The *S. aureus* strain used was USA300 JE2 (ref. 57), a laboratory-adapted strain derived from the parental USA300 strain isolated from a skin and soft tissue infection (ref. 58).

### Chemicals and reagents

All chemicals were purchased from Sigma-Aldrich unless otherwise indicated. Oligonucleotides were purchased from Integrated DNA Technologies. Wild-type and mutant calprotectins were expressed and purified as described previously^32^,^34^.

### Growth and processing of biofilms for IMS

Biofilms were grown in a Drip Flow Biofilm Reactor (DFR) (BioSurface Technologies, Bozeman, MT) similar to previously described methods^25^ using glass microscope slides as the growth surface. Glass microscope slides (VWR, USA; #48300-025) were treated overnight with filtered adult bovine plasma containing Na-EDTA (Lampire Biological Laboratories, Pipersville, PA) diluted to 20% concentration in carbonate-bicarbonate buffer and placed into the DFR chambers. Individual chambers were inoculated with 10 ml of a 1:10 dilution of an overnight bacterial culture grown in YESCA medium (10 g l^−1^ casamino acids; 1 g l^−1^ yeast extract). Cultures were incubated statically for 18 h at 37 °C before waste lines were opened and the chamber was tilted at a 10° angle to promote drainage of waste medium. Fresh YESCA medium was continuously supplied over the course of 6 days through the action of a peristaltic pump at a flow rate of ∼0.3 ml min^−1^ per chamber while incubation continued at 37 °C. At the end of the 6-day incubation, biofilms adhered to slides were removed from the DFR chambers and washed twice in 20 ml of Milli-Q water prior to subsequent processing. For calprotectin treatment of DFR biofilms, nutrient lines were detached after 5.5 days of incubation and replaced with lines containing calprotectin-treatment media (60% YESCA, 40 mM NaCl, 1.2 mM CaCl_2_, 0.5 mM β-mercaptoethanol, 8 mM Tris, pH 7.5, and 0.25 mg ml^−1^ calprotectin). The calprotectin-treatment media was supplied to the biofilms over the course of 12 h at a flow rate of ∼0.3 ml min^−1^ per chamber.

Biofilms to be cryosectioned were frozen in 25% Optimal Cutting Temperature Polymer (Tissue-Tek, SakuraFinetek, Torrance, CA). Biofilms analysed by bottom-up proteomics and ICP-MS were frozen in Milli-Q water. Biofilms were sectioned at −20 °C using a Leica CM 3050S Cryostat (Leica Microsystems, Bannockburn, IL) or a Thermo Scientific Cryostar NX70 (Thermo Fisher Scientific, Waltham, MA). Sections for MALDI IMS were cut at a thickness of 12 μm and mounted onto chilled indium-tin oxide-coated glass slides (Delta Technologies, Loveland, CO). Sections prepared for LA-ICP IMS were cut at a thickness of 35 μm and mounted onto nitric-acid washed poly(L)lysine-coated vinyl slides (Electron Microscopy Sciences, Hatfield, PA).

### Growth of agar colonies

Prior to addition of calprotectin to agar-based growth medium, the medium was cooled to 50 °C. Agar-based co-culture assays were performed on LB agar embedded with appropriate calprotectin concentrations as mentioned in the text or the equivalent volume of calprotectin buffer (100 mM NaCl, 3 mM CaCl_2_ 10 mM β-mercaptoethanol, 20 mM Tris, pH 7.5). A lawn of *S. aureus* was established on the media surface by spreading a 1:1,000 dilution of an overnight culture onto solidified media using sterile cotton swabs. Sterile discs were placed on the agar surface and inoculated with 5 μl of a 1:1,000 dilution of an overnight *P. aeruginosa* culture. Plates were incubated at 37 °C for 24 h.

Agar colonies for MALDI IMS were grown on modified ISP2 agar medium (5 g l^−1^ proteose peptone #2; 3 g l^−1^ yeast extract; 3 g l^−1^ casamino acids; 10 g l^−1^ glucose; 20 g l^−1^ agar) similar to previously described methods^59^. The medium was supplemented with appropriate calprotectin concentrations as mentioned in the text or an equivalent volume of calprotectin buffer. Ten millilitres of medium were used per 100 mm by 15 mm petri dish to create a thin layer of growth medium that is more optimal for subsequent MALDI IMS. Two microliters of a 1:1,000 dilution of an overnight *P. aeruginosa* culture were spotted onto solidified medium and plates were incubated at 37 °C for 24 h prior to MALDI IMS.

### Processing of agar colonies for MALDI IMS

Bacterial colonies were excised from the petri dish as previously described^59^ and methanol soft-landed onto a Bruker 384-well stainless steel target. A mixture of 15 mg ml^−1^ 2,5-dihydroxybenzoic acid (DHB) and 5 mg ml^−1^ α-cyano-4-hydroxycinnamic acid (CHCA) was prepared in 90% acetonitrile with 0.2% trifluoroacetic acid and sonicated until crystals were fully dissolved. Matrix was applied to sample sections using a TM-Sprayer (HTX Imaging, Carrboro, NC). The matrix was sprayed onto the sections at a flow rate of 0.2 ml min^−1^ using a pushing solvent of 90% acetonitrile. The TM-Sprayer was operated at a speed of 1,200 mm min^−1^ and at a nozzle temperature of 80 °C. The spray pattern was set to 2 mm spacing and 12 passes were applied. IMS of metabolites was collected in reflector positive ion mode on an autofleX speed mass spectrometer (Bruker Daltonics, Billerica, MA) at 400-μm spatial resolution. Fifty laser shots were acquired per pixel in random-walk mode in 10 shot steps. Data were processed using fleXimaging version 4.1.

### Processing of cystic fibrosis patient lung explants

Human lungs were obtained for research purposes from a patient with cystic fibrosis and end-stage lung disease at the time of lung transplantation. Informed consent was obtained and the protocol was approved by the Vanderbilt University Institutional Review Board. The right upper lobe was excised and frozen in 50% Optimal Cutting Temperature Polymer. The lung was sectioned at 10-μm thickness using a Leica CM 3050S Cryostat and serial sections were prepared as follows: (i) stained with H&E, (ii) Gram stained and (iii) sectioned for MALDI IMS. Optical images of H&E-stained lungs were obtained at × 20 magnification using a Leica SCN400 Brightfield Slide Scanner. For MALDI IMS analysis, biopsies from explanted cystic fibrosis lung tissues were mounted onto chilled indium-tin oxide coated glass. Tissue was sequentially washed as follows: 70% ethanol for 30 s, 100% ethanol for 30 s, 6:2:1 ethanol/chloroform/acetic acid for 2 min, 100% ethanol for 30 s, water for 30 s and 100% ethanol for 30 s^60^.

### MALDI IMS of cystic fibrosis lungs

A solution of 15 mg ml^−1^ 2,5-dihydroxyacetphenone (DHA) was prepared in 90% acetonitrile with 0.2% trifluoroacetic acid and sonicated until crystals were fully dissolved. Matrix was applied to washed sample sections using a TM-Sprayer. The matrix was sprayed onto the sections at a flow rate of 0.2 ml min^−1^ using a pushing solvent of 90% acetonitrile. The TM-Sprayer was operated at a speed of 1,200 mm min^−1^ and at a nozzle temperature of 80 °C. The spray pattern was set to 2-mm spacing and six passes were applied. The matrix coating was rehydrated using 1 ml of 50 mM acetic acid in a sealed petri dish at 85 °C for 3 min. IMS was performed using a rapifleX MALDI Tissuetyper (Bruker Daltonics). The instrument was operated in linear positive ion mode with 50 by 50 μm pixels with the laser in single beam mode. A total of 500 laser shots were collected per pixel in 50 shot increments.

### MALDI IMS of bacterial biofilms

Biofilm sections analysed by MALDI IMS were washed using sequential 30-s washes of 70, 90 and 95% ethanol. Matrix was applied as previously described for the agar colonies using only eight total passes of the robotic sprayer. IMS of biofilms was performed at 50-μm step size using a rapifleX MALDI Tissuetyper operated in linear positive ion mode with 50 by 50 μm pixels with the laser in single beam mode. A total of 500 laser shots were collected per pixel in 50 shot increments. IMS of biofilms collected at >50 μm step size was performed using an autofleX speed tandem time-of-flight mass spectrometer (Bruker Daltonics) outfitted with a Gaussian beam profile Nd:YAG laser (355 nm) and operated in a linear positive ion mode. A total of 50 laser shots were collected in random-walk mode at each pixel. Data were processed using fleXimaging version 4.1.

### Proteomics of PA14 Biofilms

Biofilms selected for bottom-up proteomics experiments were dissected into central channel and nutrient-deplete edge regions (Supplementary Fig. 5) and further dissected into four replicates prior to processing. Each region of biofilm was moved to a 15 ml conical tube for lysis and protein extraction. Cells were lysed using 1 ml of 80% acetonitrile 5% formic acid and 400 μl of bacterial protein extraction reagent (Thermo Scientific, Rockford, IL). Biofilms were homogenized using an ultrasonicator dismembrator Model 150E (Fisher Scientific). Protein was quantified using a Bradford Assay (Thermo Scientific, Rockford, IL). Twenty five micrograms of protein was removed from each sample, dried using a speedvac concentrator (Thermo Scientific), reconstituted in sample buffer and loaded onto a 10% Novex Bis-Tris Gel (Invitrogen, Carlsbad, CA) with MES Running Buffer. Samples were run into the gel at 200 V for 5 min and stained using SimplyBlue SafeStain (Invitrogen). Stained gel bands were removed and subjected to in-gel reduction, alkylation and tryptic digestion. The resulting peptides were analysed by data-dependent LC-tandem mass spectra (MS/MS) analysis as described below.

### LC-MS/MS analysis for bottom-up proteomics of PA14 biofilms

Peptides were autosampled onto a 200 mm by 0.1 mm self-packed analytical column (Jupiter 3 μm, 300A) coupled directly to an LTQ (Thermo Scientific) using a nanoelectrospray source. Peptides were eluted over a 70-min aqueous to organic gradient. A full-scan mass spectrum followed by five data-dependent MS/MS was collected with enabled dynamic exclusion to minimize the acquisition of redundant spectra. Tandem mass spectra were searched using SEQUEST against the PA14 database containing both forward and reversed versions for each entry (UniProt *P. aeruginosa* strain UCBPP-PA14, taxon 208963 reference proteome set). Identifications were filtered and compiled at the protein level using Scaffold 4 (Proteome Software) with a 5% false discovery rate (FDR) and two minimum peptide threshold. Differences in peptide enrichment in the biofilm centre versus the biofilm edge were assessed using a three-step process. First, peptides of interest were identified by assessing the peptide counts obtained from centre-derived samples versus edge-derived samples and determining a statistically-significant enrichment in a biofilm compartment using a Student's *t-*test comparing the four technical replicates of a single run. Second, this statistically-significant peptide enrichment needed to be maintained in replicate biofilms in order for the protein of interest to make the final cutoff. Finally, the averaged peptide counts of all replicate biofilm edge and centre samples needed to be significantly enriched in one of these compartments to be included in Supplementary Data 2 and 3.

### Protein identification from MALDI IMS data

Proteins of interest, selected based on unique *m/z* distributions in MALDI IMS experiments, were further purified by reversed-phase HPLC for identification. Protein extracts from the centre and edge regions of the biofilm (Supplementary Fig. 5a) were selected for protein fractionation. These samples were fractionated using a Waters 2690 Separations Module equipped with an offline fraction collector. Proteins were fractionated using a Vydac 208TP 150 mm 5 μm C8 column (Vydac Grace, Columbia, Maryland) and an aqueous to organic gradient over 120 min. Fractions were collected every minute. The separation was performed twice and fractions were combined. Fractions were dried using a Speedvac Concentrator and reconstituted in 30 μl of 40% acetonitrile. One microlitre of each well was spotted onto a MALDI anchor chip for analysis by MALDI MS. Wells containing proteins of interest were isolated and further fractionated onto Novex 16% Tricine gels and stained using SimplyBlue SafeStain. Gel bands corresponding to the mass range of interest were extracted and subjected to in-gel reduction, alkylation and tryptic digestion. The resulting peptides were sequenced as described below.

### Peptide sequencing for protein identification from IMS data

Peptides were sequenced using an Orbitrap Fusion Tribrid Mass Spectrometer (Thermo Scientific) coupled to an Easy-nLC 1000 (Thermo Fisher Scientific) ultrahigh pressure liquid chromatography system. Peptides were separated on a 75-μm inner diameter, 25-cm-long PepMap RSLC C18 column (2 μm, 100 Å, Acclaim) at a flow rate of 300 nl min^−1^ using mobile phases of 0.1% formic acid, 99.9% water (solvent A) and 0.1% formic acid, 99.9% acetonitrile (solvent B). The gradient consisted of 2–20%B in 100 min, 20–32%B in 20 min, 32–95%B in 1 min, 95%B for 4 min, 95–2%B for 2 min and the column equilibrated at 2%B for 3 min. On gradient-elution, peptides were ionized via nanoelectrospray ionization using a Nanospray Flex ion source (Thermo Fisher Scientific). The instrument was operated in a 3-s top speed data-dependent acquisition mode, where precursor ions were selected for a maximum 3-s cycle. Fourier transform mass spectra were collected at 120,000 resolution using an automated gain control (AGC) target of 200,000 and a maximum injection time of 50 ms. Precursor ions were filtered according to charge state (9>*z*>1 required) and monoisotopic precursor assignment. Previously interrogated precursor ions were excluded using a dynamic window (30 s±10 p.p.m.). Precursor ions for MS/MS analysis were isolated with a 1.5 *m/z* quadrupole mass filter isolation window. Precursor ions were fragmented with higher energy dissociation using a normalized collision energy of 35%. Ion trap MS/MS spectra were acquired using an AGC target of 1,000 and maximum injection time of 40 ms. Data analyses were performed using Protalizer software (Vulcan Analytical, Birmingham, AL). Spectra were searched against the *P. aeruginosa* strain UCBPP-PA14 UniProt database using a target FDR of 1%. Searches were performed using a 20 p.p.m. MS1 tolerance and a ±0.6 Da MS2 tolerance while allowing for up to two missed cleavages, as well as carbamidomethyation, phosphorylation and oxidation modifications.

### LA-ICP IMS

Trace element imaging was performed using an LSX-213 laser ablation system (LA, CETAC, Omaha, USA) coupled with ELEMENT 2 inductively coupled plasma mass spectrometer (Thermo Fisher Scientific, Bremen, Germany). Slide-mounted slices of biofilm were placed in a sealed ablation cell and ablated in multi-line mode (line-by-line) with a focused Nd:YAG laser beam with a spot size of 100 μm. The ablated sample particles were then online introduced to ICP-MS for the detection of isotopes of ^44^Ca^+^, ^55^Mn^+^, ^56^Fe^+^ and ^66^Zn^+^.

### Trace metal analysis for PA14 biofilms

Trace metals were quantified through ICP-MS detection from homogenates extracted for bottom-up proteomic analysis as detailed above. Samples were diluted to 10 μg of protein in 100 μl protein extraction buffer and moved into 15 ml metal-free conical tubes (VWR). Samples were digested with nitric acid by adding 1 ml of 50% HNO_3_ (Optima grade; Fisher) to the 100 μl sample and incubating at 50 °C for 18 h with caps loosened. After digestion, samples were diluted to a 10-ml final volume in Milli-Q water. Five parts per billion Ga was included in each sample as an internal standard. Elemental quantification of these samples was performed on the ELEMENT 2 inductively coupled plasma mass spectrometer coupled with an ESI autosampler (Elemental Scientific, Omaha, NE). The ICP-MS was equipped with a PFA microflow nebulizer (Elemental Scientific), a double channel spray chamber (at room temperature), a magnetic sector followed by an electric sector and a second electron multiplier. The sample uptake was achieved through self-aspiration via 0.50 mm ID sample probe and sample capillary which then introduced samples to the ICP-MS for the detection of isotopes of ^25^Mg^+^, ^44^Ca^+^, ^55^Mn^+^, ^56^Fe^+^, ^66^Zn^+^ and ^69^Ga^+^.

### Growth for RNA and metabolite extraction

Samples for metabolite analysis or RNA extraction were grown in calprotectin-treatment media (60% TSB, 40 mM NaCl, 1.2 mM CaCl_2_, 0.5 mM β-mercaptoethanol, 8 mM Tris, pH 7.5) in the presence or absence of 0.25 mg ml^−1^ calprotectin and/or 10 μM ZnCl_2_. Five millilitre cultures were grown shaking at 180 r.p.m. for 18 h at 37 °C. Cells were pelleted and retained for RNA extraction. Supernatants were retained for metabolite measurement. C.f.u. were plated to determine culture density.

### qRT–PCR and RNA-seq

Total RNA was harvested using a combination of LETS buffer (0.1 M LiCl; 10 mM EDTA; 10 mM Tris HCl pH 7.4; 1% SDS) and TRI Reagent as previously described^61^. qRT–PCR was performed using SYBR green supermix (Bio-Rad) following manufacturer's instructions using the primers listed in Supplementary Table 1. RNA samples for RNA-seq were submitted to HudsonAlpha (Huntsville, AL) for ribosomal reduction, 50 bp paired-end (PE) sequencing with 12.5 million reads per sample and subsequent data analysis. The statistical test used for this data set was a moderated *t-*test with a corrected *P* value cutoff of 0.05, asymptotic *P* value computation and Benjamini–Hochberg multiple testing correction.

At HudsonAlpha, the concentration and integrity of the total RNA was estimated by Qubit 2.0 Fluorometer (Invitrogen, Carlsbad, California) and Agilent 2100 Bioanalyzer (Applied Biosystems, Carlsbad, CA), respectively. Approximately 500 ng of total RNA was required for proceeding to downstream RNA-seq applications. First, ribosomal RNA (rRNA) was removed using Ribo-Zero Gold kit (Epicenter, Madison, WI) using manufacturer's recommended protocol. Immediately after the rRNA removal, the RNA was fragmented and primed for the first strand synthesis using the NEBnext First Strand synthesis module (New England BioLabs Inc., Ipswich, MA). Directional second strand synthesis was performed using NEBNExt Ultra Directional second strand synthesis kit. Following this, the samples were taken into standard library preparation protocol using NEBNext DNA Library Prep Master Mix Set for Illumina with slight modifications. Briefly, end-repair was done followed by polyA addition and custom adapter ligation. Post-ligated materials were individually barcoded with unique in-house Genomic Services Lab (GSL) primers and amplified through 12 cycles of PCR. Library quantity was assessed by Qubit 2.0 Fluorometer, and the library quality was estimated by utilizing a DNA 1000 chip on an Agilent 2100 Bioanalyzer. Accurate quantification of the final libraries for sequencing applications was determined using the qPCR-based KAPA Biosystems Library Quantification kit (Kapa Biosystems Inc, Woburn, MA). PE sequencing was performed using an Illumina HiSeq2500 sequencer (Illumina Inc).

### Pyocyanin measurement

Pyocyanin measurements were performed similar to previously described methods^62^. Briefly, the pyocyanin from 1 ml of supernatant obtained from samples of equivalent cell density was extracted with 0.5 ml chloroform after vigorous mixing. The chloroform layer was subsequently acidified with the addition of 0.5 ml 0.2 N HCl on vigorous mixing and the pyocyanin was eluted to the aqueous phase. Pyocyanin concentration was calculated by measuring absorbance at 520 nm.

### *In vitro* co-culture

Co-culture assays were performed in 96-well plates containing 150 μl of co-culture media (60% TSB; 40% calprotectin buffer (100 mM NaCl, 3 mM CaCl_2,_ 10 mM β-mercaptoethanol, 20 mM Tris, pH 7.5)). When noted, co-culture media was supplemented with WT or ΔS1ΔS2 (transition metal-binding deficient) calprotectin at specified concentrations in the presence or absence of specified metal concentrations. The 96-well plate co-cultures were seeded with 1:100 dilutions of the metal-limited *P. aeruginosa* and *S. aureus* mono-cultures and grown for 44 h statically at 37 °C. The metal-limited mono-cultures *P. aeruginosa* and *S. aureus* used to seed the co-cultures were first grown overnight in glucose-supplemented low nutrient broth (GLNB) (2 g l^−1^ tryptic soy broth; 2 g l^−1^ glucose) at 37 °C with shaking at 180 r.p.m. The next morning, cultures were metal-restricted by pelleting and suspending samples in Chelex 100-treated GLNB supplemented with 100 μM CaCl_2_ and 1 mM MgCl_2_. These cultures were grown at 37 °C with shaking at 180 r.p.m. for 2 h. Cultures were then pelleted, suspended in fresh metal-restricted GLNB and grown for an additional 2 h to produce metal-limited samples used for co-culture inoculation. At the completion of co-culture growth assays, samples were mixed by repeated pipetting, serially diluted in PBS and plated onto cetrimide agar and mannitol salt agar for the enumeration of *P. aeruginosa* and *S. aureus*, respectively.

Agar-based co-culture assays were performed on LB agar embedded with appropriate calprotectin concentrations as mentioned in the text or the equivalent volume of calprotectin buffer (100 mM NaCl, 3 mM CaCl_2_, 10 mM β-mercaptoethanol, 20 mM Tris, pH 7.5) and were incubated at 37 °C for 24 h.

### Growth and processing of co-culture pellicle biofilms

To obtain pellicle biofilms, a sterile glass slide was placed into a 50 ml conical tube containing 10 ml of co-culture media (60% TSB; 40% calprotectin buffer (100 mM NaCl, 3 mM CaCl_2,_ 10 mM β-mercaptoethanol, 20 mM Tris, pH 7.5)) in the presence or absence of 0.25 mg ml^−1^ calprotectin. These samples were inoculated with 10 μl of overnight cultures of PA14 and/or JE2 that had been grown in 5 ml TSB at 37 °C with shaking. Caps on pellicle cultures were left loose to allow some aeration and cultures were incubated statically for 48 h. Planktonic cells were removed by a series of three PBS washes. For bacterial enumeration, adherent cells were scraped from the slide surface, suspended in PBS, serially diluted, and plated onto MSA and TSA plates prior to colony counting. For Gram-staining, adherent cells were ethanol fixed to the slide surfaces through sequential treatments with 70, 90 and 95% ethanol prior to following standard Gram-staining procedures.

### Pyoverdine measurement

Pyoverdine measurements were performed similar to previously described methods^63^. Briefly, supernatants obtained from samples of equivalent cell density were diluted 1:10 in 10 mM Tris HCl pH 8. Two hundred microliters of sample were placed into black-walled 96-well plates (Corning Inc, Corning, NY). Using a Cytation 5 Imaging Reader (BioTek, Winooski, VT), samples were excited at 400 nm and pyoverdine fluorescence emission was measured at 447 nm.

### Microscopical analyses of biofilm

Bacterial biofilms were grown as described above and analysed by either scanning electron microscopy or confocal laser scanning microscopy as previously described^64^. Briefly, for electron microscopy, samples were fixed with 2.0% paraformaldehyde (Electron Microscopy Sciences), 2.5% gluteraldehyde (Electron Microscopy Sciences) in 0.05 M sodium cacodylate (Electron Microscopy Sciences) buffer for 24 h. After primary fixation, samples were washed three times with 0.05 M sodium cacodylate buffer before sequential dehydration with increasing concentrations of ethanol. After dehydration, samples were dried at the critical point using a Tousimis Critical Point Dryer machine, mounted onto aluminium SEM sample stubs (Electron Microscopy Sciences) and sputter-coated with 5 nm of gold-palladium. Afterward, samples were painted with a thin strip of colloidal silver (Electron Microscopy Sciences) at the edge to facilitate charge dissipation. Biofilms were imaged with an FEI Quanta 250 field-emission gun scanning electron microscope (Hillsboro, OR). For confocal microscopy, samples were stained with LIVE/DEAD BacLight bacterial viability kit which includes both Syto 9 (green) and propidium iodide (red) (Life Technologies, Carlsbad, CA) to visualize bacterial cells and calcofluor white (blue) (Sigma-Aldrich) to visualize carbohydrate capsule/matrix within the biofilm. Biofilms were mounted with ProLong Antifade (Life Technologies) and visualized with a Zeiss LSM 710 (Oberkochen, Germany). Images were analysed and both ortho and 2D renderings were generated with Zen 2010 software. Micrographs shown are representative of at least three biological replicates. Fluorescence quantification was performed as previously described^65^. Briefly, eight separate measurements were taken for each biofilm replicate in addition to two background measurements for subsequent background subtraction and analysis using Image J software. Micrographs were converted to 8-bit images and the field-of-view was selected with a rectangular selection tool. Area, mean grey value, integrated density and limit of threshold were measured using the Analyze>Measure tool within the Image J software. Background fluorescent intensity was determined by selecting a portion of the field with no appreciable cells present. Values were populated into Microsoft Excel Spreadsheet, and corrected total fluorescence (CTF) was calculated using the following equation CTF=integrated density value−(area of field × mean fluorescence of background).

### Murine model of acute pneumonia

All animal experiments were approved by the Vanderbilt Institutional Animal Care and Use Committee. The co-infection of mice was performed similar to previously published procedures for acute polymicrobial pneumonia^47^. C57BL/6 mice were obtained from Jackson Laboratories. Calprotectin-deficient (S100A9^−/−^) mice derived from a C57/BL6 background were a gift from Wolfgang Nacken (Institute of Experimental Dermatology, the University of Münster, 48149 Münster, Germany). Nine-week-old male mice were intranasally infected as previously described^66^ using 30 μl of a PBS suspension containing 1 × 10^6^ c.f.u. of both *P. aeruginosa* and *S. aureus*. Infections were allowed to progress for 38 h prior to organ harvest. Bacterial colonies were enumerated following organ homogenization and plating on both TSA and MSA agar.

### Data availability

RNA-seq data have been deposited in the Gene Expression Omnibus (GEO) database under accession code GSE81065. The mass spectrometry proteomics data have been deposited to the ProteomeXchange Consortium via the PRIDE^67^ partner repository with the data set identifier PXD004081. MALDI IMS and LA-ICP IMS data sets are not available for download due to large file size. The authors declare that all other data supporting the findings of this study are available within the article and its Supplementary Information files, or from the corresponding authors upon request.

## Additional information

**How to cite this article:** Wakeman, C. A. *et al.* The innate immune protein calprotectin promotes *Pseudomonas aeruginosa* and *Staphylococcus aureus* interaction. *Nat. Commun.* 7:11951 doi: 10.1038/ncomms11951 (2016).

## Supplementary Material

Supplementary Figures and Supplementary Table Supplementary Figures 1-13 and Supplementary Table 1

Supplementary Data 1 Total proteins identified by shotgun proteomic analyses. This table provides a complete list of all proteins identified from triplicate biofilms grown on separate days.

Supplementary Data 2 Proteins enriched in the central channel of the biofilm. This table provides a complete list of all proteins that were significantly enriched in the central channel and repressed within the nutrient-deplete edge of the biofilm. Column D indicates whether or not this enrichment occurred within untreated biofilms and column E indicates the trends identified in calprotectin-treated biofilms. All data are representative of triplicate biofilms grown and processed on separate days.

Supplementary Data 3 Proteins enriched in the nutrient-deplete edge of the biofilm. This table provides a complete list of all proteins that were significantly enriched in the nutrient- deplete edge and repressed within the central channel of the biofilm. Column D indicates whether or not this enrichment occurred within untreated biofilms and column E indicates the trends identified in calprotectin-treated biofilms. All data are representative of triplicate biofilms grown and processed on separate days.

Supplementary Data 4 Genes repressed by calprotectin exposure. A summary of an RNA-seq analysis of triplicate samples depicting all genes repressed by calprotectin exposure.

Supplementary Data 5 Genes activated by calprotectin exposure. A summary of an RNA-seq analysis of triplicate samples depicting all genes activated by calprotectin exposure.
