# Supplementary material for: The innate immune protein calprotectin promotes Pseudomonas aeruginosa and Staphylococcus aureus interaction
Source: Nat Commun. 2016 Jun 15;7:11951. doi: 10.1038/ncomms11951 (PMC4912628; doi:10.1038/ncomms11951)
Supplement: Supplementary Figures and Supplementary Table — Supplementary Figures 1-13 and Supplementary Table 1 [file ncomms11951-s1.pdf]

Supplementary figures and legends

a

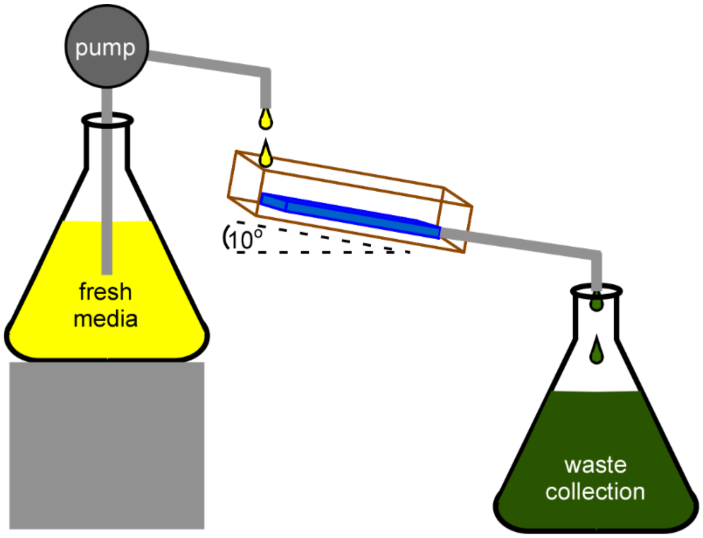

b

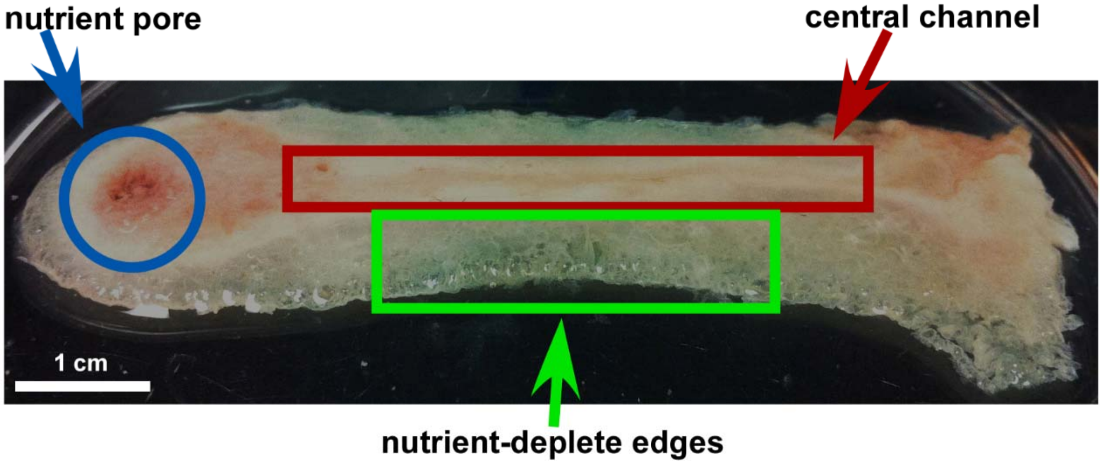

Supplementary Fig. 1: Detailed description of the structure of *P. aeruginosa* biofilms grown in a drip flow reactor (DFR). (a) Robust biofilms can be grown in a DFR, which provides a continuous influx of nutrients with minimal shear force. In this apparatus, fresh medium is supplied to bacteria in a steady drip through the action of a peristaltic pump. The medium is directed to a chamber containing a glass slide after initial seeding with bacterial culture. The chamber is placed at a 10 degree angle to allow waste to leave the chamber through gravitational action, providing minimal shear force. **(b)** A representative biofilm obtained from the DFR with overt structural features highlighted. A “nutrient pore” forms at the point where the media drips onto the glass slide (shown in blue). As the medium flows down the glass slide, a pink “central channel” forms (shown in red). Nutrients presumably diffuse out from this “central channel” to enable growth of bacteria in the “nutrient-deplete edges” (shown in green).

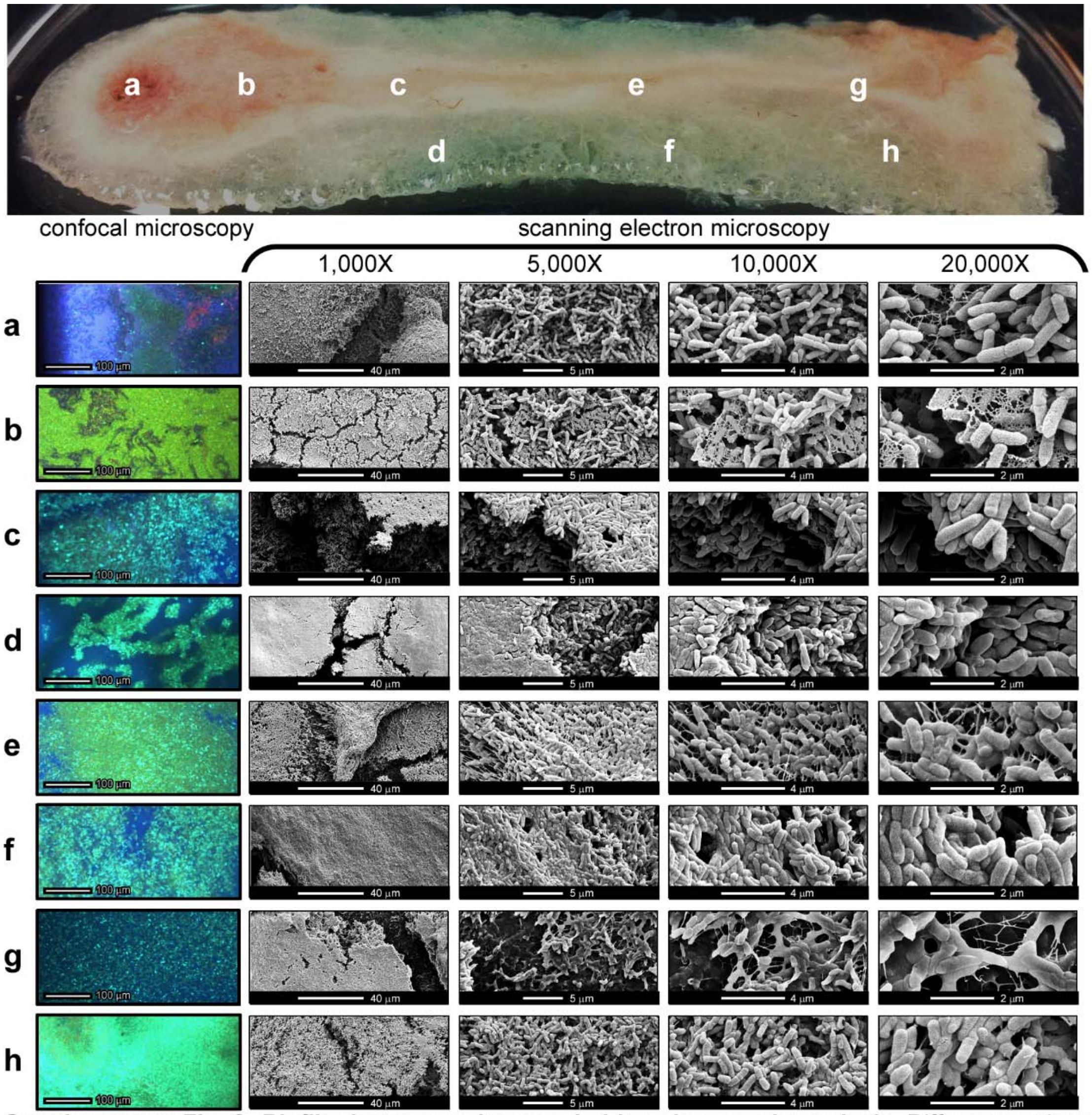

Supplementary Fig. 2: Biofilm heterogeneity revealed by microscopic analysis. Different portions of *P. aeruginosa* biofilms were analyzed by confocal and scanning electron microscopy to reveal the microscopic structural features unique to different regions of the biofilm. The letters adjacent to each picture correspond to the regions highlighted on the top biofilm image. In the confocal analysis, green stain (Syto 9) represents live cells, red stain (propidium iodide) denotes dead cells, and blue stain (calcofluor white) highlights carbohydrate-rich features. The electron micrograph images of each biofilm region are shown at four different magnifications.

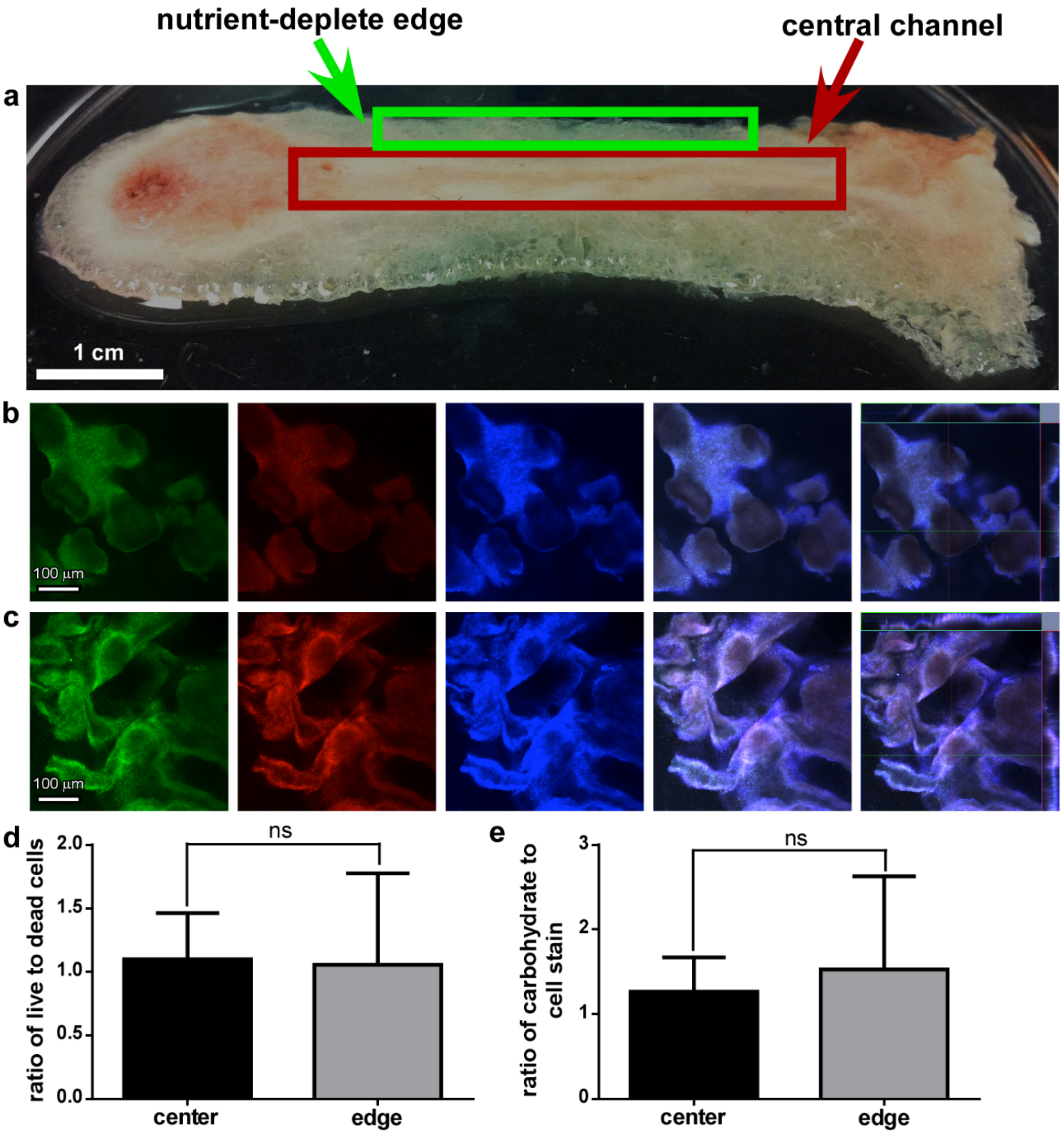

Supplementary Fig. 3: Relative quantification of live/dead stain and carbohydrate stain. Different portions of *P. aeruginosa* biofilms **(a)** were analyzed by confocal microscopy using green stain (Syto 9) to represent live cells, red stain (propidium iodide) to denote dead cells, and blue stain (calcofluor white) to highlight carbohydrate-rich features. Representative confocal images for the central channel **(b)** and the nutrient-deplete edge **(c)** are shown as separate channels, an overlay, and as an orthogonal composite. (**d)** The ratio of live to dead stain was graphed for the two major sections of the biomass. **(e)** The ratio of carbohydrate to cell stain was graphed for each section of the biomass. No significant differences were identified between the different portions of the biomass as determined by a two-tailed Student's *t*-test. Error bars represent standard deviation (*n*=4).

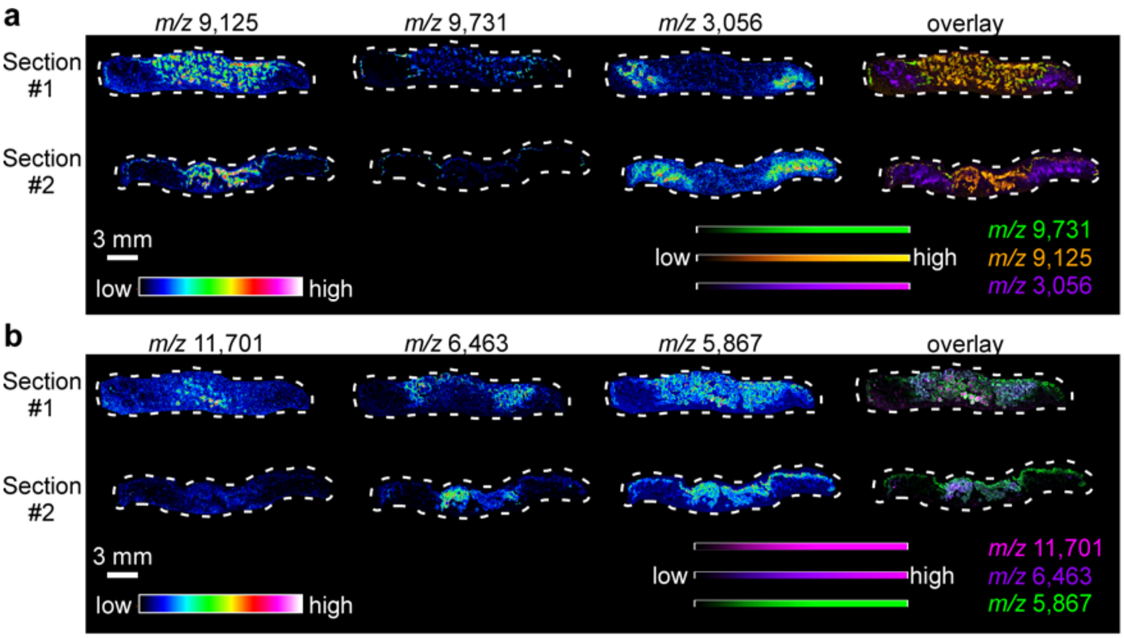

Supplementary Fig. 4: Additional heterogeneously-expressed signals reveals by MALDI IMS. Biofilm sections #1 and #2 represent the two sections most proximal to nutrient entry highlighted in Fig. 1. **(a)** A peak at *m/z* 9,731 is enriched at the interface between the nutrient-replete center (marked by *m/z* 9,125) and the nutrient-deplete edge (marked by *m/z* 3,056). **(b)** A peak at *m/z* 11,701 is found only deep within the ~3 mm thick section of the most nutrient-replete portion of the biomass whereas a peak at *m/z* 6,463 which localizes with nutrient-replete regions of the biofilm is excluded from this potentially anoxic portion. A peak at *m/z* 5,867 that might be expressed in response to high oxygen levels is found abundantly throughout the nutrient-rich portions of the biofilm but appears to be further enriched at the air-exposed surface of the biomass.

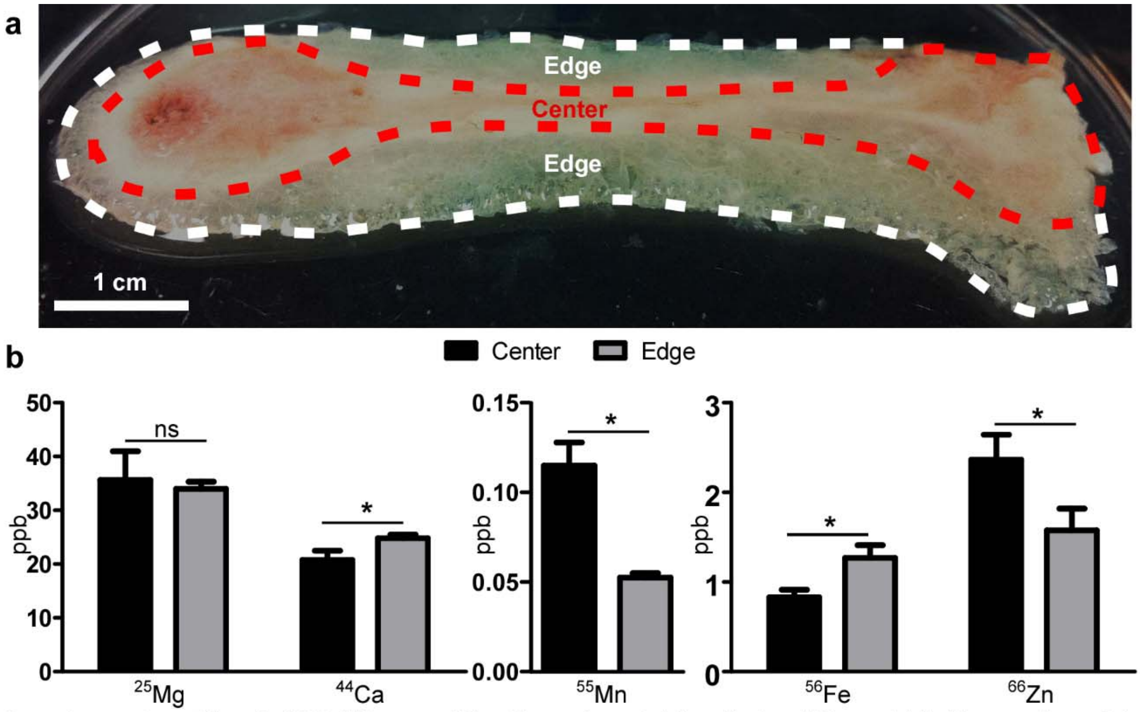

Supplementary Fig. 5: ICP-MS quantification of metal levels in different biofilm regions.(a) Approximate sections dissected from drip flow reactor biofilms for ICP-MS and proteomic analysis of the "nutrient-replete center" and "nutrient-deplete edge." **(b)** Raw ICP-MS data acquired from triplicate drip flow reactor biofilms. Biofilm homogenates were normalized to total protein content prior to analysis by ICP-MS. Error bars represent SD of triplicate samples. * denotes $p < 0.05$ as determined by a Student's *t*-test.

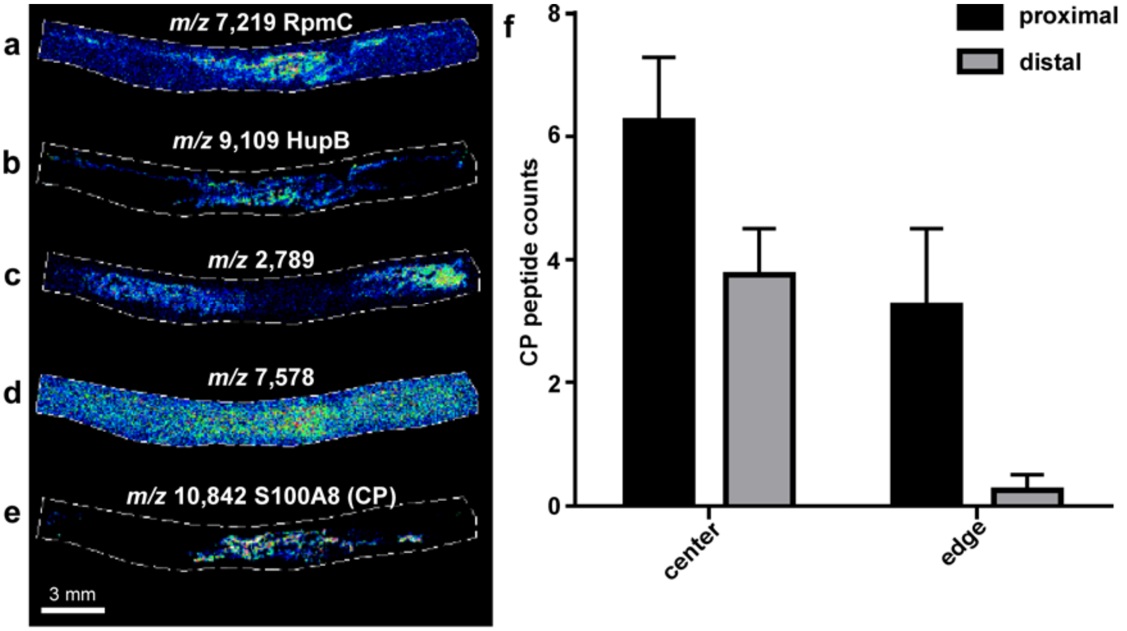

Supplementary Fig. 6: Calprotectin (CP) treatment of biofilms is incomplete, likely owing to the protective features of the biofilm structure. MALDI IMS was performed on CP-treated biofilms using 50 micron step size on an AutofleX speed mass spectrometer with CHCA/DHB matrix. These analyses revealed the presence of *m/z* signals at **(a)** 7,219 and **(b)** 9,109 identified as the central channel-localized proteins RpmC and HupB. Edge-localized and evenly-distributed signals were also observed at *m/z* of **(c)** 2,789 and **(d)** 7,578, respectively. **(e)** The signal for the S100A8 subunit of CP was primarily retained within the central channel. **(f)** Proteomic analysis of biofilm regions proximal and distal to the influx of nutrients further highlights the incomplete diffusion of CP into the distal portions of the biofilm as well as the biofilm edge as evidenced by decreased CP peptide counts. Error bars represent SEM of duplicate biofilms.

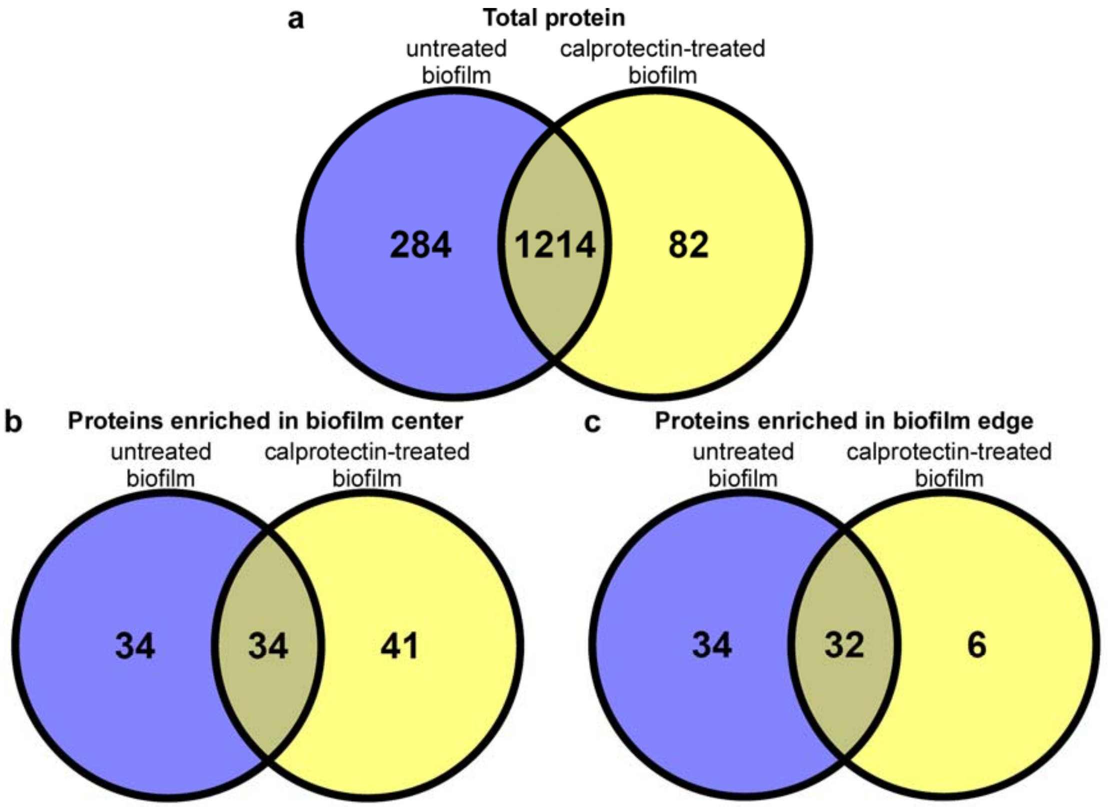

Supplementary Fig. 7: Venn diagram summary of shotgun proteomic data. (a) The total number of proteins identified from shotgun proteomics of untreated and calprotectin-treated biofilms reveals that the bulk of the proteins in both data sets are identical. **(b)** Approximately half of the proteins found to be enriched in the central channel of untreated biofilms were also enriched in the central channel of calprotectin-treated biofilms. **(c)** Approximately half of the proteins found to be enriched in the edge of untreated biofilms were also enriched in the edge of the calprotectin-treated biofilm.

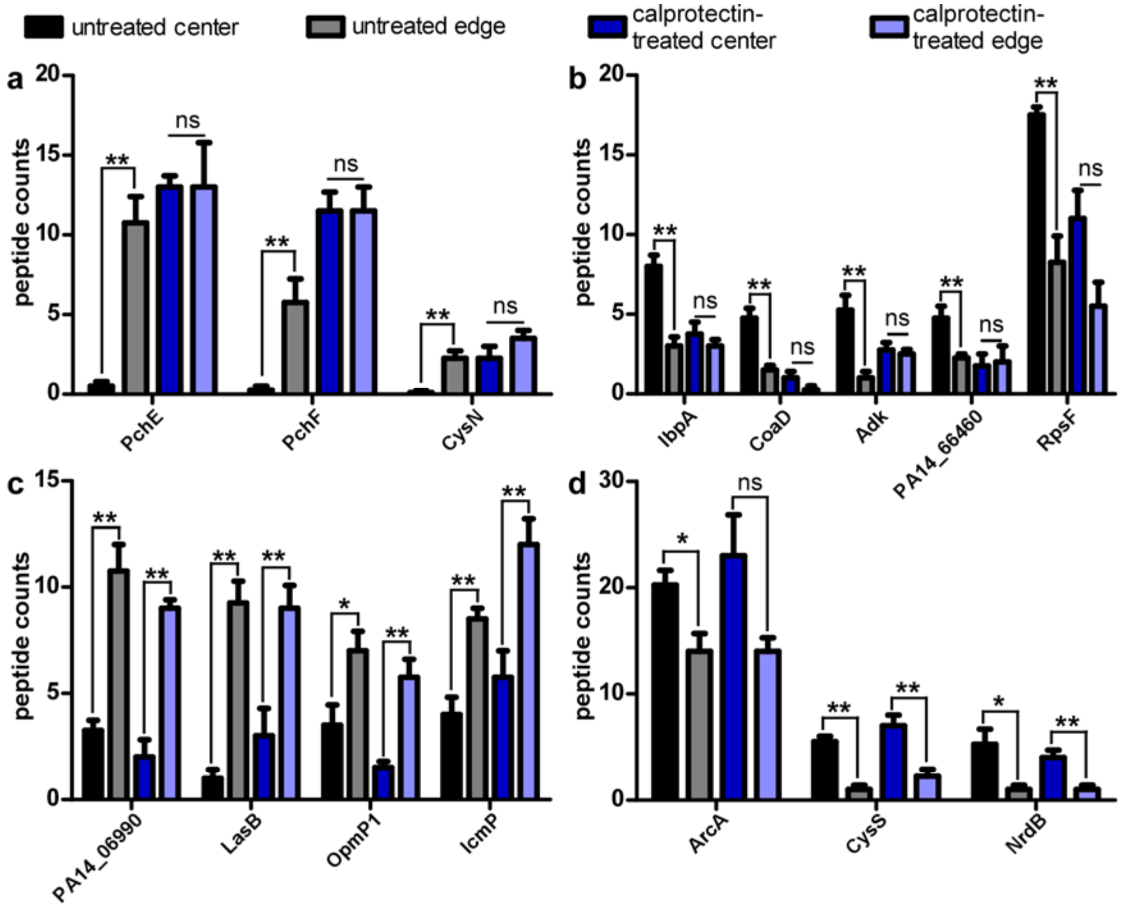

Supplementary Fig. 8: Shotgun proteomics analysis of DFR biofilms with or without calprotectin exposure reveals a subset of proteins that alter expression patterns in response to calprotectin-induced Zn and Mn deprivation. (a) Representative proteins that are activated in the biofilm edge that become constitutively activated in the presence of calprotectin. **(b)** Representative proteins that are repressed in the biofilm edge that become constitutively repressed upon calprotectin treatment. **(c)** Representative proteins activated in the biofilm edge in the presence or absence of calprotectin. **(d)** Representative proteins repressed in the biofilm edge in the presence or absence of calprotectin. Error bars denote SEM of biological triplicate samples processed in four technical replicates per biofilm. * = $p<0.05$, ** = $p<0.02$ as determined by a Student's *t*-test.

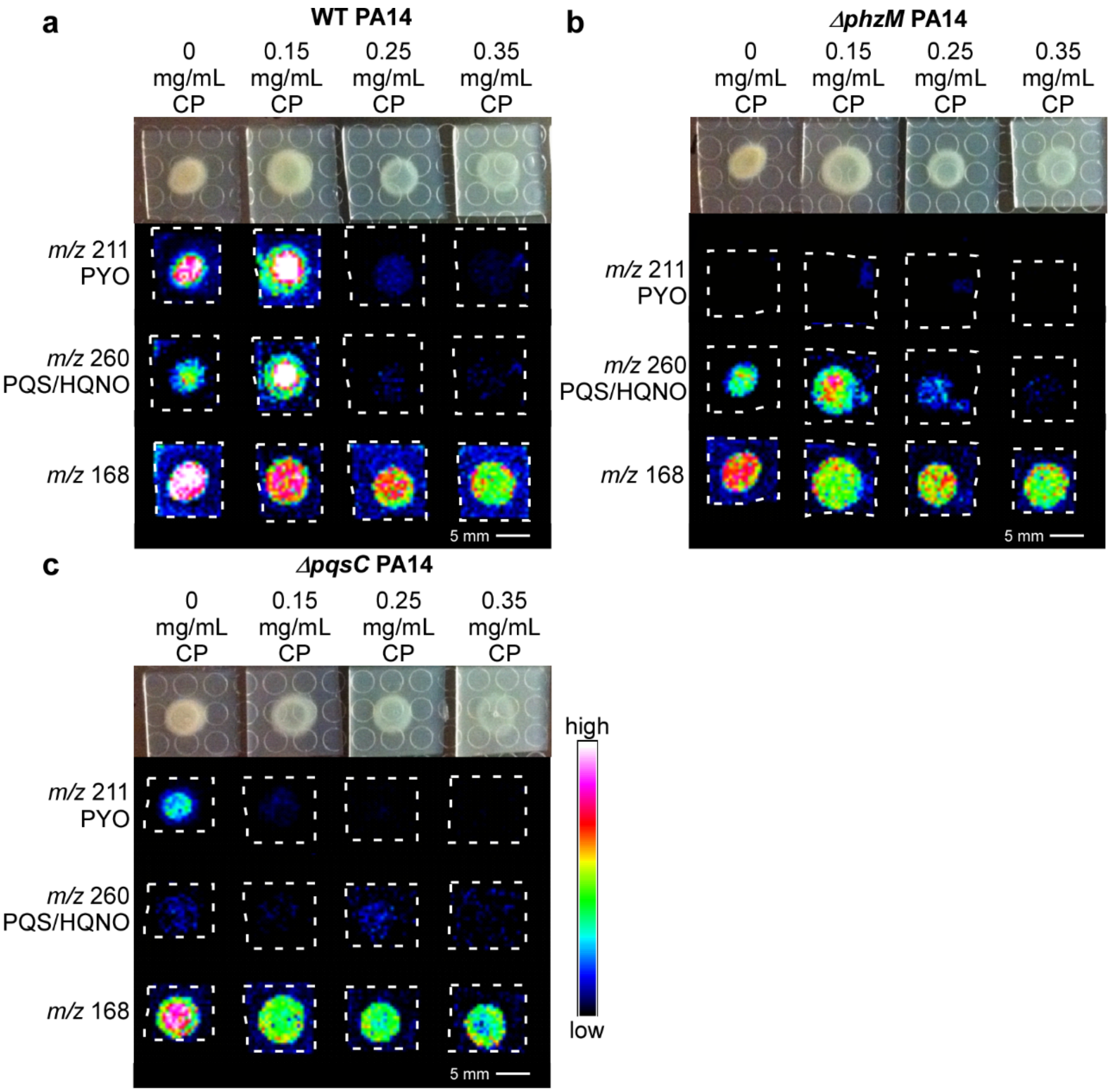

Supplementary Fig. 9: MALDI IMS comparison of WT PA14 and anti-staphylococcal factor biosynthesis mutants. (a) MALDI IMS detection of secondary metabolites, pyocyanin (PYO) and the alkyl hydroxyquinolones PQS and HQNO, as well as a control ion at *m/z* 168 on media embedded with increasing calprotectin (CP) concentrations in WT PA14. **(b)** A *ΔphzM* mutant of PA14 is unaffected in PQS/HQNO production as well as the production of the control ion; however, the pyocyanin signal is completely absent. **(c)** A *ΔpqsC* mutant of PA14 exhibits only background signal at the PQS/HQNO peak and also produces decreased levels of pyocyanin because pyocyanin production is regulated by PQS levels.

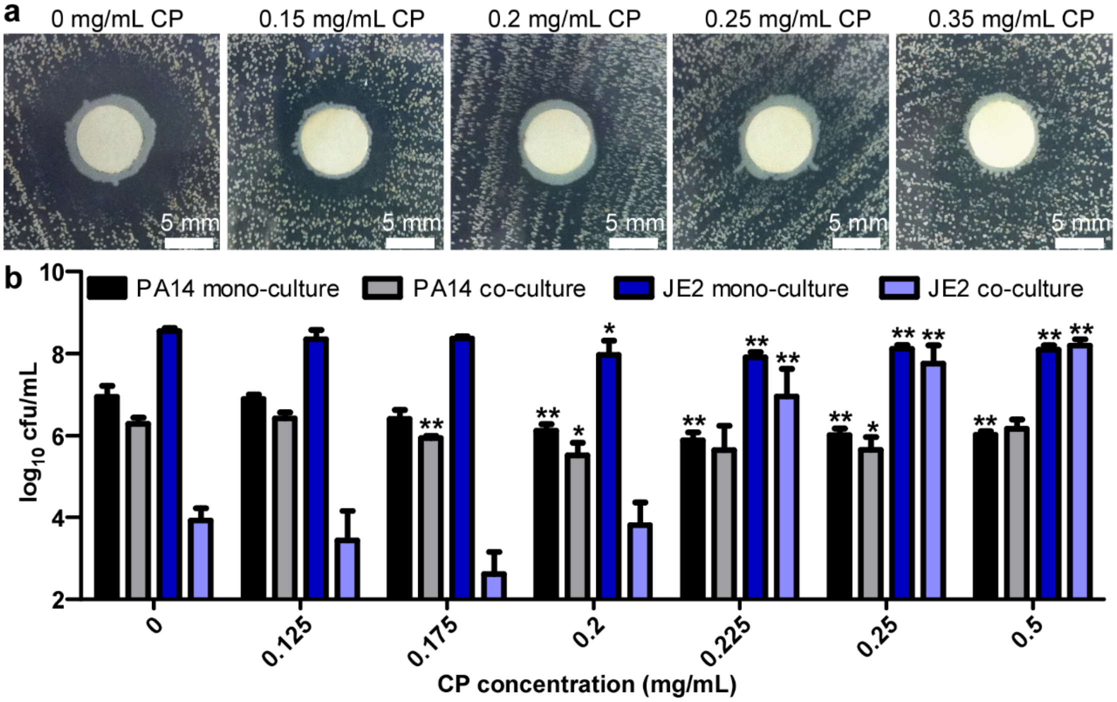

Supplementary Fig. 10: The effect of calprotectin (CP) on microbial co-culture is titratable. (a) As increasing concentrations of CP are embedded into agar-based growth media, the anti-staphylococcal activity of *P. aeruginosa* is repressed as evidenced by the reduced zone of clearing in the staphylococcal lawn around the *P. aeruginosa*-laden disk. **(b)** CFU counts of CP-exposed *S. aureus* and *P. aeruginosa* in liquid co-culture demonstrate that CP treatment represses the anti-staphylococcal ability of *P. aeruginosa* in a dose-dependent manner. Error bars represent SD of biological triplicates compiled from data generated on two separate days. * denotes $p<0.05$ and ** denotes $p<0.02$ according to a Student's *t* test compared to untreated conditions.

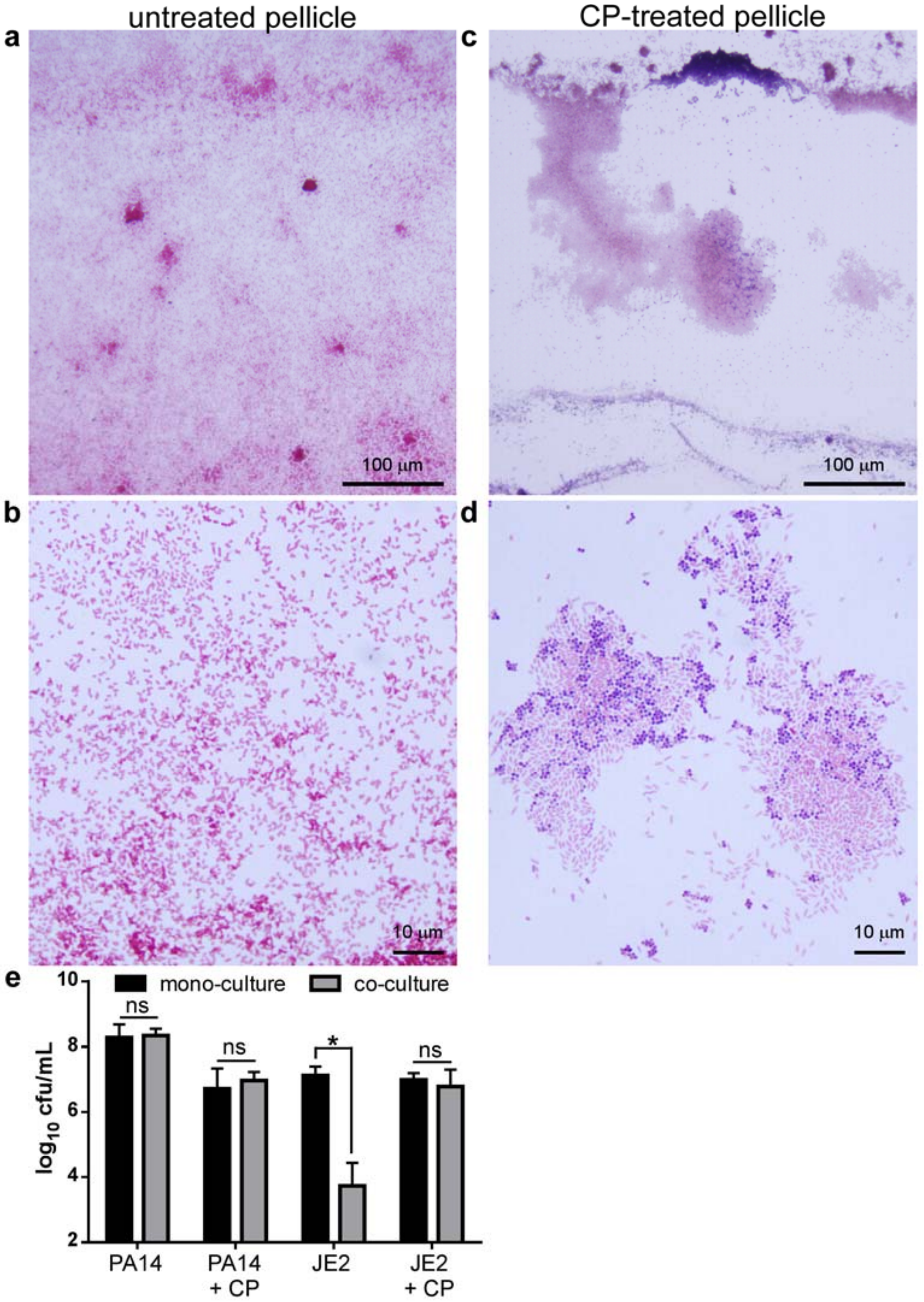

Supplementary Fig. 11: Calprotectin (CP) exposure promotes *P. aeruginosa* and *S. aureus* co-culture in pellicle biofilms. (a) Low magnification and **(b)** high magnification light microscopy of Gram-stained co-culture pellicle biofilms grown in the absence of CP reveal no detectable presence of *S. aureus*. **(c)** Low magnification and **(d)** high magnification light microscopy of Gram-stained co-culture pellicle biofilms grown in the presence of 0.25 mg/mL CP reveal significant co-culture of *P aeruginosa* (pink rods) and *S. aureus* (purple spheres). **(e)** CFU enumeration confirms that *P. aeruginosa* and *S. aureus* co-culture significantly increases in the presence of CP. Error bars represent standard deviation of seven biological replicates. * denotes $p<0.05$ as determined by a two-tailed Student's *t*-test.

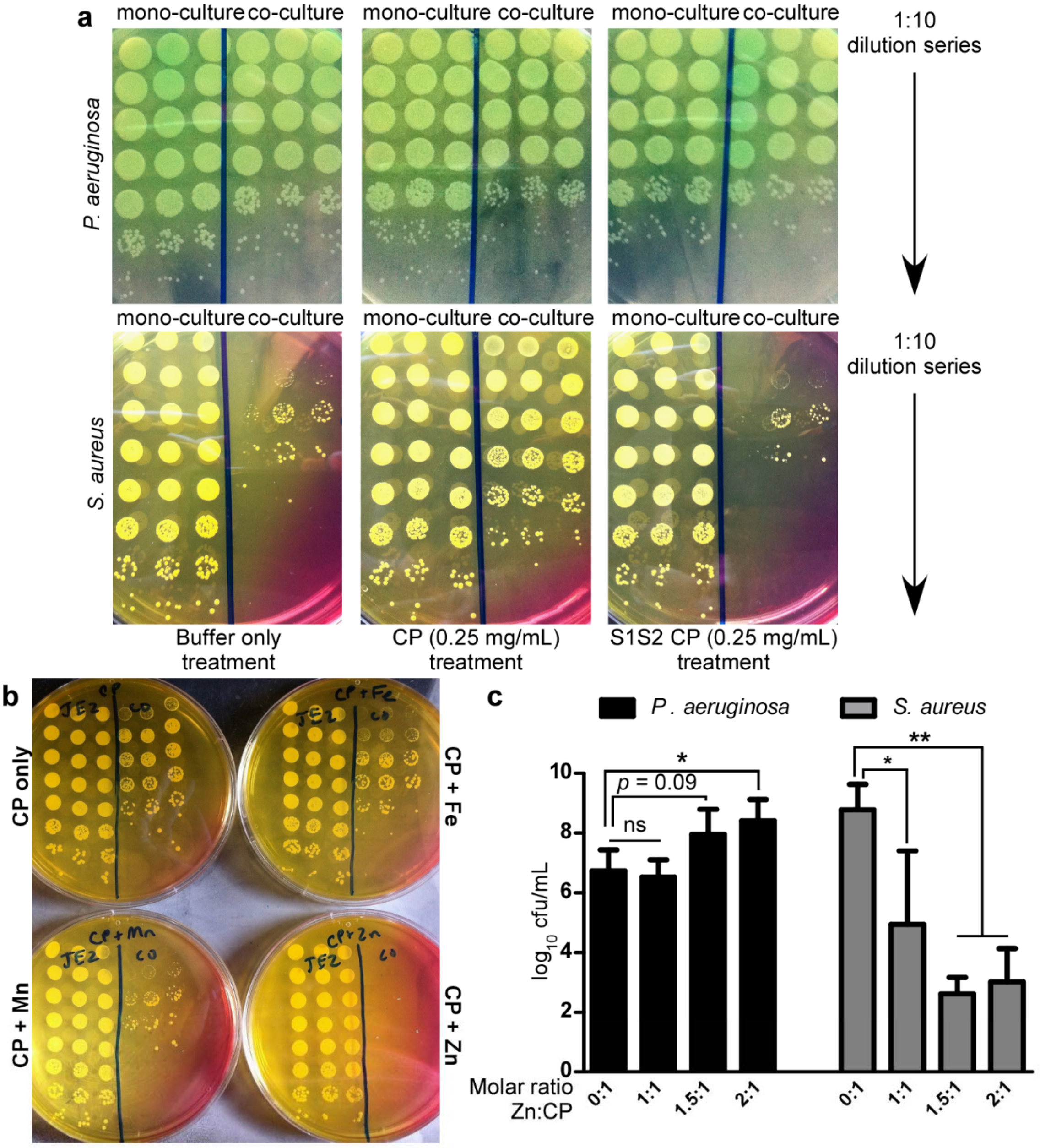

Supplementary Fig. 12: Repression of the *P. aeruginosa* anti-staphylococcal activity by calprotectin (CP) exposure is independent of CP-induced growth inhibition and is dependent on CP-mediated Zn chelation. **(a)** Representative cfu enumeration following mono- or co-culture in the presence or absence of CP with minimal difference observed in *P. aeruginosa* colony numbers but a dramatic increase in viable *S. aureus* observable upon CP exposure. In the presence of a mutant CP incapable of metal binding (S1S2 CP), cultures behave identically to buffer-treated cells. **(b)** Multiple transition metals were tested at a 10 μM concentration for the ability to reverse the protective phenotype of CP exposure on co-cultured *S. aureus* cells. The addition of Zn was the only metal to completely restore the anti-staphylococcal activity of *P. aeruginosa.* Biological triplicates of mono-culture exposure are in columns labeled "JE2" and biological triplicates of co-culture exposure are in columns labeled "co." **(c)** Quantification of replicate cfu data obtained on at least 3 separate days with and without Zn added at 1:1 molar ratio with CP (6.85 μM $ZnCl_2$), 1.5:1 molar ratio (10 μM $ZnCl_2$), or 2:1 molar ratio (13.7 μM $ZnCl_2$) to saturate the 2 Zn binding sites of CP. Analyses were performed in biological triplicate on each day. Error bars represent SD. * = $p<0.05$ and ** = $p<0.001$ as determined by a Student's *t*-test.

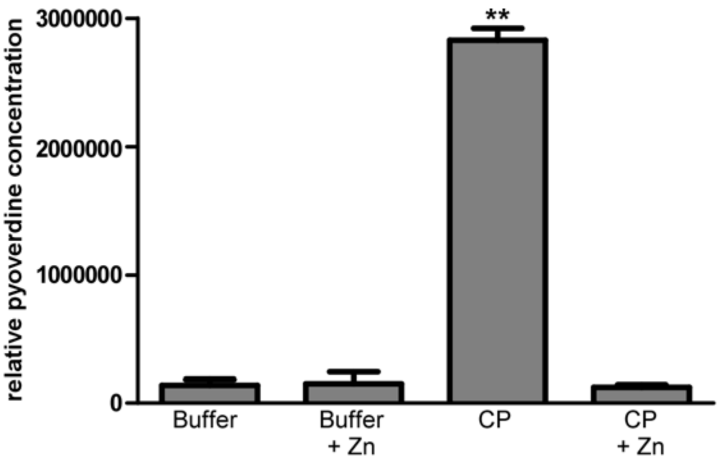

Supplementary Fig. 13: Calprotectin (CP)-induced pyoverdine production is reversed by Zn addition. Quantification of pyoverdine in cultures grown in the presence or absence of 0.25 mg/mL CP. The activation of pyoverdine production in the presence of CP is reversed by the addition of 10 μM Zn. ** designates p<0.002 as determined by a Student's *t* test. Error bars represent SD of triplicate samples.

Supplementary Table 1: Primers for qRT-PCR

Primer target	Forward primer	Reverse primer
phzA1/2	AACCACTTCTGGGTCGAGTG	GTGGGAATACCGTCACGTTT
hcnB	ATCTCAAGCTCAACGGCATT	GTATCCGTCGTCGCTGAAG
pqsH	ATGTCTACGCGACCCTGAAG	AACTCCTCGAGGTCGTTGTG
lasA	GGAGCGGCTACTACAGCATC	CTGGCGCAACTGATATTCCT
**16S rRNA**	AAGCAACGCGAAGAACCTTA	CACCGGCAGTCTCCTTAGAG

Supplementary data legends

Supplementary Data 1: Total proteins identified by shotgun proteomic analyses. This table provides a complete list of all proteins identified from triplicate biofilms grown on separate days.

Supplementary Data 2: Proteins enriched in the central channel of the biofilm. This table provides a complete list of all proteins that were significantly enriched in the central channel and repressed within the nutrient-deplete edge of the biofilm. Column D indicates whether or not this enrichment occurred within untreated biofilms and column E indicates the trends identified in calprotectin-treated biofilms. All data are representative of triplicate biofilms grown and processed on separate days.

Supplementary Data 3: Proteins enriched in the nutrient-deplete edge of the biofilm. This table provides a complete list of all proteins that were significantly enriched in the nutrient-deplete edge and repressed within the central channel of the biofilm. Column D indicates whether or not this enrichment occurred within untreated biofilms and column E indicates the trends identified in calprotectin-treated biofilms. All data are representative of triplicate biofilms grown and processed on separate days.

Supplementary Data 4: Genes repressed by calprotectin exposure. A summary an RNA-seq analysis of triplicate samples depicting all genes repressed by calprotectin exposure.

Supplementary Data 5: Genes activated by calprotectin exposure. A summary an RNA-seq analysis of triplicate samples depicting all genes activated by calprotectin exposure.
